# Losartan in hospitalized patients with COVID-19 in North America: An individual participant data meta-analysis

**DOI:** 10.1097/MD.0000000000033904

**Published:** 2023-06-09

**Authors:** Leon Di Stefano, Malathi Ram, Daniel O. Scharfstein, Tianjing Li, Preeti Khanal, Sheriza N. Baksh, Nichol McBee, Charles D. Bengtson, Anne Gadomski, Matthew Geriak, Michael A. Puskarich, Matthias A. Salathe, Aletta E. Schutte, Christopher J. Tignanelli, Jennifer Victory, Barbara E. Bierer, Daniel F. Hanley, Daniel A. Freilich

**Affiliations:** a Department of Biostatistics, Johns Hopkins Bloomberg School of Public Health, Baltimore, MD; b Department of International Health, Johns Hopkins Bloomberg School of Public Health, Baltimore, MD; c Division of Brain Injury Outcomes, Johns Hopkins School of Medicine, Baltimore, MD; d Division of Biostatistics, Department of Population Health Sciences, University of Utah School of Medicine, Salt Lake City, UT; e University of Colorado Denver, Anschutz Medical Campus, Denver, CO; f Johns Hopkins Bloomberg School of Public Health, Baltimore, MD; g Department of Internal Medicine, University of Kansas Medical Center, KS City, KS; h Bassett Research Institute, Bassett Medical Center, Cooperstown, NY; i Department of Research, Sharp Healthcare, San Diego, CA; j Department of Emergency Medicine, University of Minnesota, Minneapolis, MN; k Department of Emergency Medicine, Hennepin County Medical Center, Minneapolis, MN; l School of Population Health, University of New South Wales, The George Institute for Global Health, Sydney, NSW, Australia; m Department of Surgery, University of Minnesota, Minneapolis, MN; n Department of Medicine, Brigham and Women’s Hospital, Boston, MA; o Harvard Medical School, Boston, MA; p Department of Internal Medicine, Division of Infectious Diseases, Bassett Medical Center, Cooperstown, NY.

**Keywords:** angiotensin receptor antagonists, angiotensin-converting enzyme inhibitors, COVID-19, individual participant data meta-analysis, losartan, SARS-CoV-2

## Abstract

Angiotensin-converting enzyme inhibitors and angiotensin II receptor blockers (ARBs) have been hypothesized to benefit patients with COVID-19 via the inhibition of viral entry and other mechanisms. We conducted an individual participant data (IPD) meta-analysis assessing the effect of starting the ARB losartan in recently hospitalized COVID-19 patients.

**Methods:**

We searched ClinicalTrials.gov in January 2021 for U.S./Canada-based trials where an angiotensin-converting enzyme inhibitors/ARB was a treatment arm, targeted outcomes could be extrapolated, and data sharing was allowed. Our primary outcome was a 7-point COVID-19 ordinal score measured 13 to 16 days post-enrollment. We analyzed data by fitting multilevel Bayesian ordinal regression models and standardizing the resulting predictions.

**Results:**

325 participants (156 losartan vs 169 control) from 4 studies contributed IPD. Three were randomized trials; one used non-randomized concurrent and historical controls. Baseline covariates were reasonably balanced for the randomized trials. All studies evaluated losartan. We found equivocal evidence of a difference in ordinal scores 13-16 days post-enrollment (model-standardized odds ratio [OR] 1.10, 95% credible interval [CrI] 0.76–1.71; adjusted OR 1.15, 95% CrI 0.15–3.59) and no compelling evidence of treatment effect heterogeneity among prespecified subgroups. Losartan had worse effects for those taking corticosteroids at baseline after adjusting for covariates (ratio of adjusted ORs 0.29, 95% CrI 0.08–0.99). Hypotension serious adverse event rates were numerically higher with losartan.

**Conclusions:**

In this IPD meta-analysis of hospitalized COVID-19 patients, we found no convincing evidence for the benefit of losartan versus control treatment, but a higher rate of hypotension adverse events with losartan.

## 1. Introduction

Renin-angiotensin system inhibitors (RASi), especially angiotensin-converting enzyme inhibitors (ACEi) and angiotensin II receptor blockers (ARB), are commonly used classes of medications for hypertension, proteinuric kidney disease, heart failure, and acute coronary syndrome. RASi are well tolerated, though side effects of hyperkalemia, renal dysfunction, and hypotension can occur. Research into RASi for treatment of COVID-19 was recommended, in part, based on amelioration of SARS-CoV-induced pulmonary histopathologic perturbations in animal models.^[1–3]^ Retrospective-observational studies comparing exposure versus non-exposure and continuation versus discontinuation, and open-label trials of ACEi/ARB in COVID-19 patients, produced equivocal evidence around efficacy and safety, with some suggesting benefit.^[4–16]^ Blinded, placebo-controlled randomized clinical trials (RCTs), on the other hand, have not shown efficacy but have yielded concerning safety signals.^[17–19]^

We conducted an individual participant data (IPD) meta-analysis of trials from the USA and Canada with the goal of assessing the efficacy and safety of initiating ACEi/ARB in hospitalized COVID-19 patients and to examine whether ACEi/ARB efficacy might vary among patient subgroups. This investigation was adapted from a similar IPD meta-analysis of trials of hydroxychloroquine/chloroquine for hospitalized patients with COVID-19 conducted by the Trial Innovation Network and COVID-19 Collaboration Platform.^[20]^

## 2. Methods

### 2.1. Study selection and protocol

We searched ClinicalTrials.gov on January 26, 2021, using the terms “COVID-19,” “angiotensin-converting enzyme,” “ACE,” “angiotensin receptor blockers,” and “ARB.” Trial inclusion criteria were: hospitalized COVID-19 patients; treatment with new prescription of ACEi or ARB vs control (placebo, standard of care, other); consent forms and/or trial institutional review boards allowed sharing of individual-level data; trialists agreed to preplanned data harmonization, analysis, extraction/upload, and sharing plans; and outcome measurements were collected or extractable. The Mary Imogene Bassett Hospital Institutional Review Board (Cooperstown, NY) approved the study and determined that the study’s secondary research, using exclusively deidentified data, was exempt from Institutional Review Board review according to federal regulations (Exempt Category 4) (#1773896-1; June 24, 2021). The study protocol was registered with the International Prospective Register of Systematic Reviews (PROSPERO; CRD42021267770).^[21]^

### 2.2. Data collection and harmonization

A common data harmonization tool, including a data dictionary with definitions and encodings of variables, example data, and de-identification functions for dates and ages consistent with US Health Insurance Portability and Accountability Act requirements, was used by trial teams to create data sets that were uploaded 1773896-1 to the data repository Vivli (https://www.vivli.org) and then downloaded for our meta-analysis. We resolved queries about missing, unusual, or inconsistent data via direct contact with study trialists and, in some cases, manual chart review (see Table S1, Supplemental Digital Content, http://links.lww.com/MD/J42, which demonstrates the study’s data dictionary).

### 2.3. Primary, secondary, and safety outcomes

The primary outcome and key secondary outcomes were based on a modified version of the 7-level ordinal outcome score used in the Adaptive COVID-19 Treatment Trial.^[22]^ The score is defined as follows: death; hospitalized, on invasive mechanical ventilation or extracorporeal membrane oxygenation; hospitalized, on noninvasive ventilation or high flow oxygen; hospitalized, requiring supplemental oxygen; hospitalized, not requiring supplemental oxygen; not hospitalized, limitation on activities; and not hospitalized, no limitations on activities. Levels 6 and 7 of the scale were merged. The ordinal score for a given time interval was taken as the earliest available measurement in that interval. Treatment effects for the ordinal score were quantified using cumulative odds ratios. This scale is relatively coarse compared with others in use (for example the 11-point WHO scale),^[23]^ and was chosen to make the data easier to harmonize.

The primary outcome was the ordinal score between days 13 and 16. Secondary outcomes were the ordinal score measured at 7 days and between 28–30 days post-enrollment, as well as mortality between 13–16 and between 28–30 days post-enrollment, hospitalization length of stay, and duration of mechanical ventilation. Mortality outcomes were compared using risk differences, while distributions of length of stay and mechanical ventilation were compared in an exploratory manner.

Safety outcomes were overall adverse event (AE) and serious adverse event (SAE) rates, and the rates of specific AEs and SAEs of interest (acute kidney injury [AKI], hyperkalemia, and hypotension), as defined in each study’s safety reporting procedures. Due to practical constraints, no attempt was made to harmonize these definitions across studies.

### 2.4. Baseline and post-baseline variables

From each trial we requested individual-level treatment assignment and baseline covariates including age (binned in 5-year intervals and truncated at age 90), sex, race and ethnicity, body mass index, number of symptomatic days before enrollment, baseline corticosteroid use, and baseline ordinal score.

We also requested patients’ status for the following comorbidity variables (all coded as yes/no): AIDS, cerebrovascular disease, prior myocardial infarction, congestive heart failure, dementia, chronic obstructive pulmonary disease, asthma, history of hypertension, HIV status (without AIDS), solid tumor, liver disease, diabetes mellitus, cigarette or tobacco smoking, and vaping.

### 2.5. Statistical analysis: primary outcome

For our analysis of the ordinal and mortality outcomes, we fit a Bayesian multilevel proportional odds ordinal regression model. The model adjusted for the individual-level covariates sex, age, baseline ordinal score, number of baseline comorbidities (truncated at 4), baseline corticosteroid use, and symptom onset days before enrollment; it included random effects for study and treatment-by-study interactions. Fixed effect coefficients, including the treatment effect, were given uniform priors, and random effect standard deviations were modeled as independently half Student t test with 3 degrees of freedom and scale parameter 2.5. Ordinal cut points were modeled as Student t distributed with 3 degrees of freedom and scale parameter 2.5 subject to a monotonicity constraint. We fit the model using R (version 4.04) and the library “brms” (version 2.14).^[24]^ We imputed missing baseline covariates using multiple imputation by chained equations, as implemented in the R package “mice” (version 3.12)^[25]^; the posterior was pooled across 10 imputations.

From this model we produced 2 effect estimates: a model-standardized (marginal) cumulative odds ratio (OR; our primary measure of effect), and an adjusted (conditional) cumulative OR. The model-standardized estimate was formed by averaging the predicted outcome probabilities from the fitted model, under treatment and under control, over the empirical distribution of individual-level covariates in the pooled population. From these average predicted outcomes, we computed the geometric mean of cumulative ORs over the 5 cut points of the modified ordinal outcome score; this corresponds to the cumulative OR when the proportional odds assumption holds. The adjusted cumulative OR, on the other hand, was estimated using the coefficient for treatment in the regression model. We also computed a “plug-in” estimate, which we produced by fitting a proportional odds model to the outcome data with treatment as the sole covariate using maximum likelihood (implemented in polr from the R package “MASS,” version 7.3-53).^[26]^

### 2.6. Statistical analysis: subgroup and interaction effects

To examine subgroup and interaction effects, we fit a model with the same structure as above but augmented with treatment-covariate interactions for the following prespecified covariates: age, numeric baseline ordinal score, baseline corticosteroids, and symptom onset days before enrollment. As in the primary analysis, we produced 2 kinds of estimates: model-standardized subgroup estimates and adjusted (interaction) estimates. We also computed subgroup plug-in estimates.

For subgroup effects, we split the pooled study population into covariate-based bins. For continuous covariates (age and symptoms onset days before enrollment) we divided the study population based on empirical tertiles. We also conducted a subgroup analysis based on a “baseline risk score” given by each individual’s expected linear predictor under control (averaged over study effects).

### 2.7. Secondary and safety outcomes

We analyzed secondary outcomes involving the ordinal score at different time-points in the same way as the primary outcome. We analyzed mortality outcomes using the same models as were fit to the corresponding ordinal score outcomes, but here our effect measure was the risk difference; we also computed “plug-in” analogues. We analyzed other secondary and safety outcomes descriptively.

### 2.8. Model checking and sensitivity analysis

We assessed the adequacy of our statistical models using posterior predictive checks and residual plots, and cross validation-based model diagnostics using the R package “loo,” version 2.4.1.^[27]^

### 2.9. Changes to statistical analysis plan following data collection

We modified our prespecified statistical analysis plan (SAP) following data harmonization, sample size determination, and examination of missingness in baseline and outcome variables. The most significant SAP changes (made before analyzing outcomes) were changing our outcome window from day 28–30 to day 13–16 post-enrollment; merging levels 6 and 7 of the ordinal score; and simplifying our prespecified statistical model. The first 2 changes were motivated by missingness in the outcome data and differing follow-up protocols among the included studies. The third change was motivated by the smaller than expected sample size and a desire to simplify the statistical model following our experience with an earlier IPD meta-analysis of hydroxychloroquine/chloroquine.^[20]^ see Table S2, Supplemental Digital Content, http://links.lww.com/MD/J43, which summarizes these and other SAP changes.

### 2.10. Risk of bias assessment

Two investigators independently assessed risk of bias associated with the effect of treatment assignment on the primary outcome using the Risk of Bias 2 tool^[28]^ for the 3 randomized trials and the Risk Of Bias In Non-randomized Studies of Interventions (ROBINS-I) tool^[29]^ for the one non-randomized study, with disagreements resolved through discussion. Since we analyzed IPD, we excluded the fifth domain “risk of bias in selection of the reported result.” We followed the recommended algorithms to reach an overall “risk of bias” assessment for each study.

See Methods, Supplemental Digital Content, http://links.lww.com/MD/J53 and Supplemental PRISMA IPD-MA, Supplemental Digital Content, http://links.lww.com/MD/J54, which provide additional details on the study’s methods and a PRISMA IPD-MA checklist, respectively).

## 3. Results

### 3.1. Study characteristics

**Figure 1.**
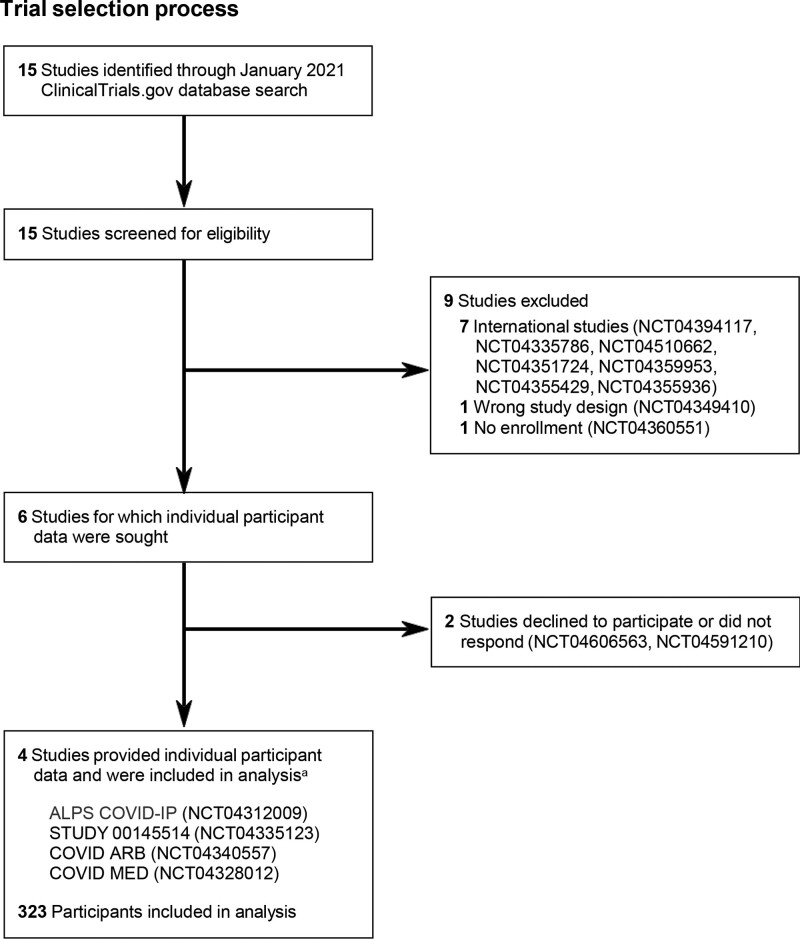
Trial selection process. Full study names are as follows: ALPS COVID-IP, Losartan for Patients With COVID-19 Requiring Hospitalization; COVID ARB, Do Angiotensin Receptor Blockers Mitigate Progression to Acute Respiratory Distress Syndrome With SARS-CoV-2 Infection?; COVID MED, Comparison Of Therapeutics for Hospitalized Patients Infected With SARS-CoV-2 In a Pragmatic aDaptive randoMizED Clinical Trial During the COVID-19 Pandemic; STUDY 00145514, Study of Open Label Losartan in COVID-19.

The initial search yielded 15 trials. We excluded trials outside of the US and Canada because of international data sharing requirements (e.g., General Data Protection Regulation); this reduced the list to 8 trials. Two investigators (D.F. and M.R.) reviewed ClinicalTrials.gov study summaries on February 11, 2021, and May 3, 2021, as well as documents provided by trialists (i.e., protocols, consent forms, and data dictionaries) to confirm eligibility. Four trials met inclusion criteria and agreed to participate (Fig. 1): ALPS-COVID IP, sponsored by University of Minnesota (NCT04312009, n = 205); STUDY 00145514, sponsored by University of Kansas (NCT04335123, n = 77); COVID ARB, sponsored by Sharp HealthCare (NCT04340557, n = 31); and COVID MED, sponsored by Bassett Medical Center (NCT04328012, n = 12). The trials were not published prior to the search, although 3 of the 4 have been published since.^[14,15,19]^

The ARB losartan was the treatment arm in all 4 studies; dosing was typically 25–50 mg orally daily up to 100 mg/d to complete a 14-day course or until discharge. No other ARB and no ACEi were evaluated in any of the studies.

Three of the 4 studies were RCTs. In two of these trials, the control arm was placebo; in one, it was usual care. The fourth study, STUDY 00145514, was a single-arm trial augmented with both nonrandomized concurrent and historical controls. In their own analysis, STUDY 00145514 investigators matched patients in the control arm to those in the losartan arm using propensity scores. We were able to obtain IPD on all the treated and control patients, but could not obtain data on which controls were included in the matched dataset. Because of this, we decided to conduct our analyses both including and excluding STUDY 00145514 data.

See Table S3, Supplemental Digital Content, http://links.lww.com/MD/J44, which provides further information about the study’s characteristics (primary, secondary, and safety outcomes). Also see Table S4, Supplemental Digital Content, http://links.lww.com/MD/J45, which provides further information about the study’s characteristics (treatment groups, participant assessment, and inclusion/exclusion criteria).

### 3.2. Risk of bias assessment

In the randomized studies, we assessed 2 studies (ALPS-COVID IP, COVID ARB) as being at low risk of bias in the first domain of “Bias arising from the randomization process.” COVID MED scored “high risk of bias” due to group-specific exclusion criteria that was determined after randomization. All randomized studies scored low for “Bias due to deviations from the intended intervention” and “Bias due to missing outcome data.” Two studies (ALPS-COVID IP, COVID MED) scored low in “Bias in the measurement of the outcome.” COVID ARB scored high in this domain as it was open-label. Overall, two of the randomized studies (COVID ARB, COVID MED) had high risk of bias. STUDY 00145514, the non-randomized study, scored moderate risk of bias for “Bias due to confounding” for inadequate control of time-varying confounders and serious risk of bias for “Bias in selection of participants into the study” for selection of participants after the start of the intervention. Overall, STUDY 00145514 had serious risk of bias. See Table S5, Supplemental Digital Content, http://links.lww.com/MD/J46, which summarizes risk of bias assessment across outcomes for each study.

### 3.3. Patient characteristics and missing data

A total of 325 hospitalized patients (156 losartan vs 169 controls) with laboratory-confirmed SARS-CoV-2 infection and meeting pooling criteria were included in the study population. Enrollment occurred at a median of 7 days post-symptoms onset (interquartile range 4–9), with 55% of patients in the losartan arms initiating dosing on the enrollment day. Almost all patients (97%) were enrolled within 2 days of hospitalization.

Key baseline demographic characteristics were reasonably balanced between the pooled losartan and control populations. For the losartan and control groups, respectively, mean age was 53 years versus 54 years, male sex was 59% versus 59%, white race was 43% versus 37%, and median body mass index 32 versus 31. Of the patients assigned to losartan, 44% were taking corticosteroids at baseline, compared with 40% in the control group. However, clinical characteristics were unbalanced between the losartan and control groups. There were disparities in the percentage of patients supported by noninvasive ventilation (ordinal score 3 [hospitalized, noninvasive ventilation]: 21% vs 14%) and in the percentage of patients without any supportive oxygen (ordinal score 5 [hospitalized, no oxygen]: 17% vs 27%). There were also disparities in the number of days between hospital admission and enrollment (enrolled on day of admission: 21% losartan vs 49% control; enrolled 1 day after admission: 53% vs 36%; enrolled 2 days after admission: 22% vs 13%).

This imbalance was driven by STUDY 00145514, the non-RCT among our included studies. The pool of controls from STUDY 00145514 had less severe disease at baseline than the losartan group (ordinal score 2 [hospitalized, mechanical ventilation]: 13% vs 9%; score 3 [hospitalized, noninvasive ventilation]: 17% vs 2%; score 4 [hospitalized, supplemental oxygen]: 50% vs 32%; score 5 [hospitalized, no oxygen]: 20% vs 57%), and all control patients had their outcomes recorded from the day of hospital admission. This imbalance motivated sensitivity analyses excluding patients from STUDY 00145514; in particular, “plug-in” estimates from the pooled population are likely biased. See Table 1 for further details.

**Table 1:** Participant characteristics overall and by study.

	Overall		Randomized trials only (excluding STUDY 00145514)		ALPS-COVID IP		STUDY 00145514		COVID ARB		COVID MED	
	Losartan (N = 156)	Control (N = 169)	Losartan (N = 126)	Control (N = 122)	Losartan (N = 101)	Control (N = 104)	Losartan (N = 30)	Control (N = 47)*	Losartan (N = 16)	Control (N = 15)	Losartan (N = 9)	Control (N = 3)
Sex, n (%)												
Female	64 (41)	70 (41)	51 (40)	48 (39)	42 (42)	41 (39)	13 (43)	22 (47)	6 (38)	6 (40)	3 (33)	1 (33)
Male	92 (59)	99 (59)	75 (60)	74 (61)	59 (58)	63 (61)	17 (57)	25 (53)	10 (62)	9 (60)	6 (67)	2 (67)
Race, n (%)												
American Indian/Alaska Native	0 (0)	1 (1)	0 (0)	1 (1)	0 (0)	1 (1)	0 (0)	0 (0)	0 (0)	0 (0)	0 (0)	0 (0)
Asian	8 (5)	4 (2)	7 (6)	2 (2)	7 (7)	2 (2)	1 (3)	2 (4)	0 (0)	0 (0)	0 (0)	0 (0)
Black/African American	45 (29)	52 (31)	38 (30)	30 (25)	37 (37)	30 (29)	7 (23)	22 (47)	1 (6)	0 (0)	0 (0)	0 (0)
Native Hawaiian/Pacific Islander	1 (1)	0 (0)	0 (0)	0 (0)	0 (0)	0 (0)	1 (3)	0 (0)	0 (0)	0 (0)	0 (0)	0 (0)
White	67 (43)	62 (37)	46 (37)	51 (42)	35 (35)	47 (45)	21 (70)	11 (23)	2 (12)	1 (7)	9 (100)	3 (100)
Multiple	0 (0)	0 (0)	0 (0)	0 (0)	0 (0)	0 (0)	0 (0)	0 (0)	0 (0)	0 (0)	0 (0)	0 (0)
Other/declined	12 (8)	24 (14)	12 (10)	13 (11)	0 (0)	0 (0)	0 (0)	11 (23)	12 (75)	13 (87)	0 (0)	0 (0)
Unknown/unavailable	23 (15)	26 (15)	23 (18)	25 (20)	22 (22)	24 (23)	0 (0)	1 (2)	1 (6)	1 (7)	0 (0)	0 (0)
Ethnicity, n (%)												
Hispanic	44 (28)	49 (29)	33 (26)	36 (30)	20 (20)	23 (22)	11 (37)	13 (28)	12 (75)	13 (87)	1 (11)	0 (0)
Not Hispanic	108 (69)	112 (66)	89 (71)	79 (65)	80 (79)	76 (73)	19 (63)	33 (70)	3 (19)	1 (7)	6 (67)	2 (67)
Unavailable	4 (3)	8 (5)	4 (3)	7 (6)	1 (1)	5 (5)	0 (0)	1 (2)	1 (6)	1 (7)	2 (22)	1 (33)
Age (5-year bins)												
Median (IQR)	50 (40–65)	55 (45–65)	50 (40–65)	55 (45–60)	50 (40–60)	55 (45–60)	50 (40–65)	55 (45–65)	52 (42–76)	50 (40–65)	70 (55–70)	60 (58–65)
Body mass index												
Median (IQR)	32 (28–37)	31 (27–35)	32 (28–36)	31 (26–36)	32 (28–38)	32 (27–37)	33 (28–39)	30 (27–33)	30 (28–34)	28 (26–33)	32 (29–33)	38 (33–39)
Missing, n (%)	0 (0)	2 (1)	0 (0)	1 (1)	0 (0)	1 (1)	0 (0)	1 (2)	0 (0)	0 (0)	0 (0)	0 (0)
Baseline ordinal score, n (%)												
2: hosp, mech vent	11 (7)	12 (7)	7 (6)	8 (7)	6 (6)	8 (8)	4 (13)	4 (9)	0 (0)	0 (0)	1 (11)	0 (0)
3: hosp, NIV	33 (21)	23 (14)	28 (22)	22 (18)	23 (23)	21 (20)	5 (17)	1 (2)	0 (0)	0 (0)	5 (56)	1 (33)
4: hosp, supp oxygen	85 (54)	89 (53)	70 (56)	74 (61)	56 (55)	58 (56)	15 (50)	15 (32)	13 (81)	14 (93)	1 (11)	2 (67)
5: hosp, no oxygen	27 (17)	45 (27)	21 (17)	18 (15)	16 (16)	17 (16)	6 (20)	27 (57)	3 (19)	1 (7)	2 (22)	0 (0)
Baseline ordinal score (numeric)												
Median (IQR)	3 (2–3)	3 (3–4)	3 (2–3)	3 (3–3)	3 (2–3)	3 (2–3)	3 (2–3)	4 (3–4)	3 (3–3)	3 (3–3)	2 (2–3)	3 (2–3)
Days between hospital admission and enrollment, n (%)												
0 (enrolled on day of admission)	33 (21)	83 (49)	29 (23)	36 (30)	29 (29)	35 (34)	4 (13)	47 (100)*	0 (0)	1 (7)	0 (0)	0 (0)
1 day	83 (53)	60 (36)	67 (53)	60 (49)	56 (55)	51 (49)	16 (53)	0 (0)*	6 (38)	7 (47)	5 (56)	2 (67)
2 days	34 (22)	22 (13)	24 (19)	22 (18)	16 (16)	17 (16)	10 (33)	0 (0)*	5 (31)	5 (33)	3 (33)	0 (0)
≥3 days	6 (4)	4 (2)	6 (5)	4 (3)	0 (0)	1 (1)	0 (0)	0 (0)*	5 (31)	2 (13)	1 (11)	1 (33)
Days between symptom onset and enrollment												
Median (IQR)	7 (5–9)	7 (4–9)	7 (5–9)	7 (4–9)	7 (5–9)	7 (4–9)	8 (6–10)	6 (3–7)	6 (4–8)	6 (4–8)	NA	NA
Missing, n (%)	14 (9)	13 (8)	12 (10)	8 (7)	3 (3)	4 (4)	2 (7)	5 (11)	0 (0)	1 (7)	9 (100)	3 (100)
Baseline comorbidity count												
Median (IQR)	1 (0–1)	1 (0–2)	1 (0–1)	1 (0–1)	1 (0–1)	1 (0–1)	1 (0–2)	1 (0–2)	1 (0–1)	0 (0–1)	0 (0–1)	3 (2–3)
Missing, n (%)	5 (3)	2 (1)	5 (4)	2 (2)	4 (4)	2 (2)	0 (0)	0 (0)	0 (0)	0 (0)	1 (11)	0 (0)
Corticosteroid use at baseline, n (%)												
Yes	69 (44)	68 (40)	68 (54)	67 (55)	60 (59)	65 (62)	1 (3)	1 (2)	0 (0)	0 (0)	8 (89)	2 (67)
No	87 (56)	101 (60)	58 (46)	55 (45)	41 (41)	39 (38)	29 (97)	46 (98)	16 (100)	15 (100)	1 (11)	1 (33)
Days between enrollment and first dose, n (%)												
0 (first dose received on day of enrollment)	86 (55)	33 (20)‡	58 (46)	33 (27)‡	36 (36)	31 (30)	28 (93)	NA†	16 (100)	NA†	6 (67)	2 (67)
1 day	66 (42)	65 (38)‡	64 (51)	65 (53)‡	62 (61)	64 (62)	2 (7)	NA†	0 (0)	NA†	2 (22)	1 (33)
2 days	0 (0)	1 (1)‡	0 (0)	1 (1)‡	0 (0)	1 (1)	0 (0)	NA†	0 (0)	NA†	0 (0)	0 (0)
Missing	4 (3)	70 (41)‡	4 (3)	23 (19)‡	3 (3)	8 (8)	0 (0)	47 (100)†	0 (0)	15 (100)†	1 (11)	0 (0)
Missing outcome score (d13-d16), n (%)												
Yes	22 (14)	33 (20)	1 (1)	1 (1)	1 (1)	1 (1)	21 (70)	32 (68)	0 (0)	0 (0)	0 (0)	0 (0)
No	134 (86)	136 (80)	125 (99)	121 (99)	100 (99)	103 (99)	9 (30)	15 (32)	16 (100)	15 (100)	9 (100)	3 (100)
Missing outcome score (d13-d16; levels 6/7 merged), n (%)												
Yes	1 (1)	1 (1)	1 (1)	1 (1)	1 (1)	1 (1)	0 (0)	0 (0)	0 (0)	0 (0)	0 (0)	0 (0)
No	155 (99)	168 (99)	125 (99)	121 (99)	100 (99)	103 (99)	30 (100)	47 (100)	16 (100)	15 (100)	9 (100)	3 (100)

IQR = interquartile range, NIV = noninvasive ventilation (includes BiPAP/CPAP and/or high-flow oxygen).

* Data are from a combination of historical and nonrandomized concurrent controls; outcome data were collected from the first day of hospitalization.

† Control arm was standard care.

‡ These subgroups include patients assigned to standard care.

Several comorbidity indicators had substantial missingness (tumor, dementia, cerebrovascular disease, AIDS, liver disease, vaping, and smoking) and thus were excluded from the comorbidity count covariate in our statistical models.

### 3.4. Primary outcome – main analysis

**Figure 2.**
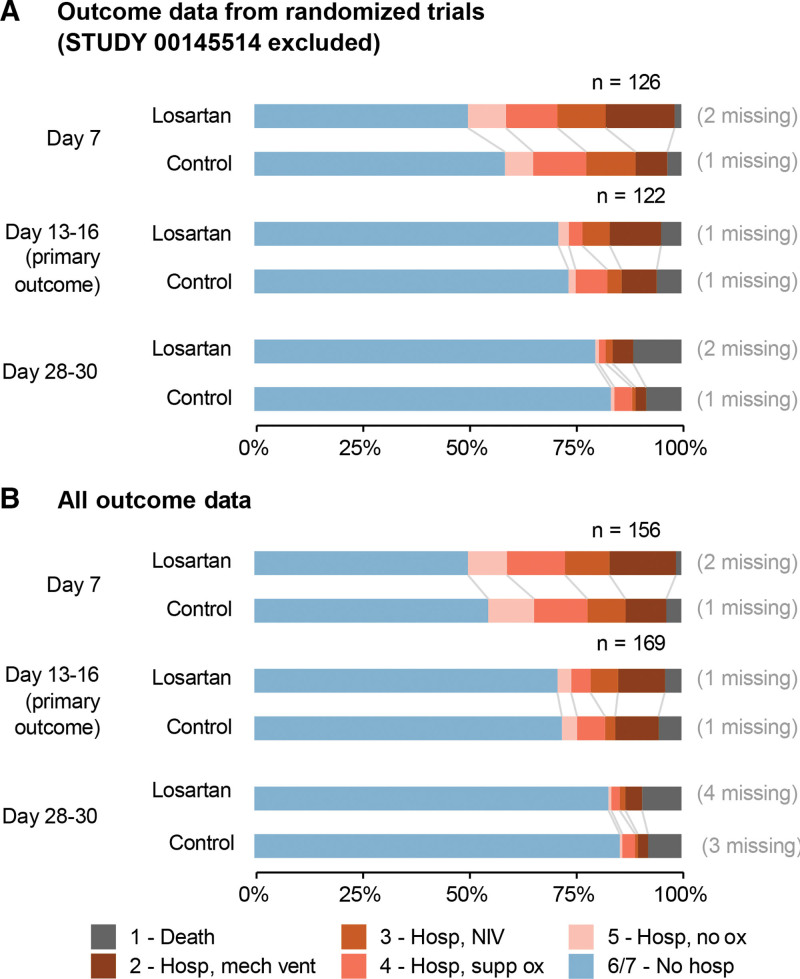
(A) Ordinal outcome data from the randomized trials only (ALPS-COVID IP, COVID ARB, and COVID MED; STUDY 00145514 data excluded). (B) All ordinal outcome data, including data from STUDY 00145514. Deaths have been carried forward; other outcome missingness is indicated on the right side of the figure. Values of n indicate number of patients in each arm at enrollment.

The standardized proportional OR for the primary outcome, ordinal COVID score 13–16 days after enrollment in the pooled study population, was 1.10 (95% credible interval [CrI] 0.76–1.71; higher values favor ACEi/ARB); the covariate-adjusted OR was 1.15 (95% CrI 0.15–3.59) and unadjusted plug-in proportional OR was 0.96 (95% confidence interval [CI] 0.59–1.54). These results are consistent with no effect for ACEi/ARB treatment on COVID-19 ordinal score. Results were similar for other choices of time-points (day 7 and days 28–30 post-enrollment). See Figure 2 and Table 2.

**Table 2:** Primary, secondary, and safety outcomes, overall and by study.

		Overall (N = 325)		Randomized trials only (excluding STUDY 00145514) (N = 248)		ALPS-COVID IP (N = 205)		STUDY 00145514 (N = 77)		COVID ARB (N = 31)		COVID MED (N = 12)	
Ordinal score at d13-16	Standardized OR (95% CrI)	1.10 (0.76–1.71)		0.89 (0.58–1.34)*		0.93 (0.57–1.59)		1.56 (0.84–4.05)		1.68 (0.59–9.37)		1.07 (0.34–2.68)	
	Plug-in OR (95% CI)	0.96 (0.59–1.54)		0.88 (0.50–1.52)		0.90 (0.49–1.65)		1.21 (0.47–3.30)		2.59 (0.43–21.08)		NA†	
	Conditional OR (95% CrI)	1.15 (0.15–3.59)		0.75 (0.02–3.46)*		0.91 (0.47–1.71)		1.86 (0.76–5.94)		1.77 (0.61–9.96)		1.04 (0.17–3.69)	
Ordinal score at d7	Standardized OR (95% CrI)	0.87 (0.63–1.22)		0.65 (0.40–0.95)*		0.66 (0.42–1.00)		1.59 (0.78–3.44)		1.62 (0.54–5.54)		1.00 (0.27–2.73)	
	Plug-in OR (95% CI)	0.81 (0.53–1.21)		0.71 (0.44–1.14)		0.66 (0.39–1.11)		1.13 (0.48–2.65)		1.67 (0.36–8.22)		0.27 (0.01–3.13)	
	Conditional OR (95% CrI)	0.99 (0.16–3.53)		0.61 (0.05–2.67)*		0.58 (0.33–1.04)		1.90 (0.75–5.15)		1.60 (0.51–6.57)		0.99 (0.18–4.36)	
Ordinal score at d28-30	Standardized OR (95% CrI)	0.92 (0.52–1.61)		0.76 (0.45–1.31)*		0.81 (0.46–1.56)		1.97 (0.55–16.65)		1.23 (0.28–8.83)		0.69 (0.12–2.13)	
	Plug-in OR (95% CI)	0.82 (0.44–1.49)		0.76 (0.40–1.46)		0.88 (0.44–1.76)		2.76 (0.38–55.63)		NA†		NA†	
	Conditional OR (95% CrI)	0.76 (0.02–3.32)		0.52 (0.01–2.45)*		0.75 (0.34–1.58)		2.20 (0.52–26.07)		1.18 (0.23–11.07)		0.65 (0.06–3.02)	
Mortality at d13-16	Standardized RD (95% CrI)	0.00 (−0.02 to 0.03)		−0.01 (−0.04 to 0.02)*		0.00 (−0.03 to 0.02)		0.02 (−0.01 to 0.07)		0.01 (−0.01 to 0.04)		0.01 (−0.13 to 0.11)	
	Plug-in RD (95% CI)	0.01 (−0.04 to 0.07)		0.01 (−0.05 to 0.07)		0.00 (−0.07 to 0.06)		0.04 (−0.04 to 0.13)		0.07 (−0.12 to 0.26)		0.00 (NA)‡	
Mortality at d28-30	Standardized RD (95% CrI)	−0.01 (−0.05 to 0.04)		−0.02 (−0.08 to 0.03)*		−0.02 (−0.08 to 0.04)		0.03 (−0.03 to 0.08)		0.01 (−0.06 to 0.06)		−0.06 (−0.33 to 0.12)	
	Plug-in RD (95% CI)	−0.01 (−0.08 to 0.05)		−0.03 (−0.11 to 0.05)		−0.02 (−0.11 to 0.07)		0.07 (−0.04 to 0.17)		0.00 (−0.17 to 0.18)		−0.25 (−0.78 to 0.28)	
		**Losartan (N** = **156**)	**Control (N** = **169**)	**Losartan (N** = **126**)	**Control (N** = **122**)	**Losartan (N** = **101**)	**Control (N** = **104**)	**Losartan (N** = **30**)	**Control (N = 47**)	**Losartan (N = 16**)	**Control (N = 15**)	**Losartan (N = 9**)	**Control (N = 3**)
Hospitalization LoS (median, in days)		7	6	7	6	7	6	9	7	4	3	14	7
Patients on mechanical ventilation between enrollment and d28, n (% of nonmissing)		32 (21)	27 (16)	26 (21)	17 (14)	21 (21)	17 (17)	6 (20)	10 (21)	1 (6)	0 (0)	4 (50)	0 (0)
All events	AEs (n, rate per patient)	161 (1.03)	263 (1.56)	79 (0.63)	66 (0.54)	55 (0.54)	60 (0.58)	82 (2.73)	197 (4.19)	7 (0.44)	6 (0.4)	17 (1.89)	0 (0)
	SAEs (n, rate per patient)	69 (0.44)	43 (0.25)	69 (0.55)	43 (0.35)	50 (0.5)	42 (0.4)	0 (0)	0 (0)	1 (0.06)	1 (0.07)	18 (2)	0 (0)
AKI events	AEs (n, rate per patient)	31 (0.2)	40 (0.24)	21 (0.17)	19 (0.16)	20 (0.2)	19 (0.18)	10 (0.33)	21 (0.45)	0 (0)	0 (0)	1 (0.11)	0 (0)
	SAEs (n, rate per patient)	2 (0.01)	2 (0.01)	2 (0.02)	2 (0.02)	2 (0.02)	2 (0.02)	0 (0)	0 (0)	0 (0)	0 (0)	0 (0)	0 (0)
Hyperkalemia events	AEs (n, rate per patient)	8 (0.05)	13 (0.08)	4 (0.03)	4 (0.03)	3 (0.03)	3 (0.03)	4 (0.13)	9 (0.19)	0 (0)	1 (0.07)	1 (0.11)	0 (0)
	SAEs (n, rate per patient)	0 (0)	0 (0)	0 (0)	0 (0)	0 (0)	0 (0)	0 (0)	0 (0)	0 (0)	0 (0)	0 (0)	0 (0)
Hypotension events	AEs (n, rate per patient)	27 (0.17)	24 (0.14)	14 (0.11)	9 (0.07)	6 (0.06)	4 (0.04)	13 (0.43)	15 (0.32)	7 (0.44)	5 (0.33)	1 (0.11)	0 (0)
	SAEs (n, rate per patient)	17 (0.11)	6 (0.04)	17 (0.13)	6 (0.05)	14 (0.14)	6 (0.06)	0 (0)	0 (0)	0 (0)	0 (0)	3 (0.33)	0 (0)

* Model-based estimates in the “Randomized trials only” column are based on a model fit only to data from those trials (i.e., excluding data from STUDY 00145514).

† The plug-in proportional odds model could not be fit within these subgroups.

‡ There were no deaths in the COVID MED population by d13-16.

### 3.5. Primary outcome – subgroup and interaction analyses

**Figure 3.**
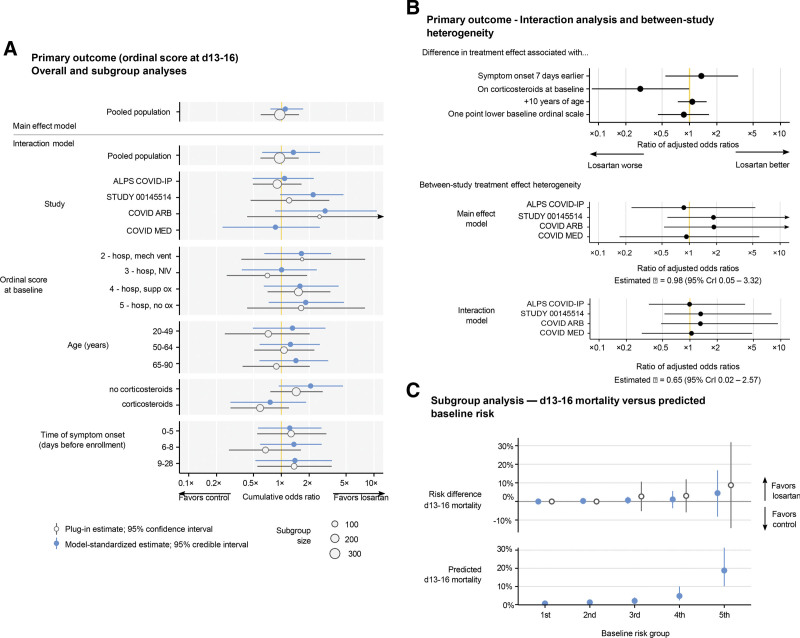
Subgroup and interaction analyses. (A) Subgroup analysis comparing plug-in and model-standardized estimates. Plug-in estimates are given with 95% confidence intervals. Model-standardized estimates are posterior medians with 95% credible intervals. Subgroup estimates are from the model including treatment-covariate interactions; an estimate in the pooled population using the main effect-only model is shown for comparison. (B) Interaction analysis and between-study heterogeneity. Estimates are posterior means with 95% credible intervals. (C) Subgroup analysis for mortality based on baseline risk. Risk groups are based on quintiles of expected outcomes under control in the model with treatment-covariate interactions. Plug-in estimates are given with 95% confidence intervals; model-standardized estimates are posterior medians with 95% credible intervals.

Our subgroup analysis found no appreciable effects of losartan within any prespecified subgroup (see Fig. 3A and Table S6, Supplemental Digital Content, http://links.lww.com/MD/J47, which demonstrates subgroup effects for day 13–16 ordinal score and mortality).

**Figure 4.**
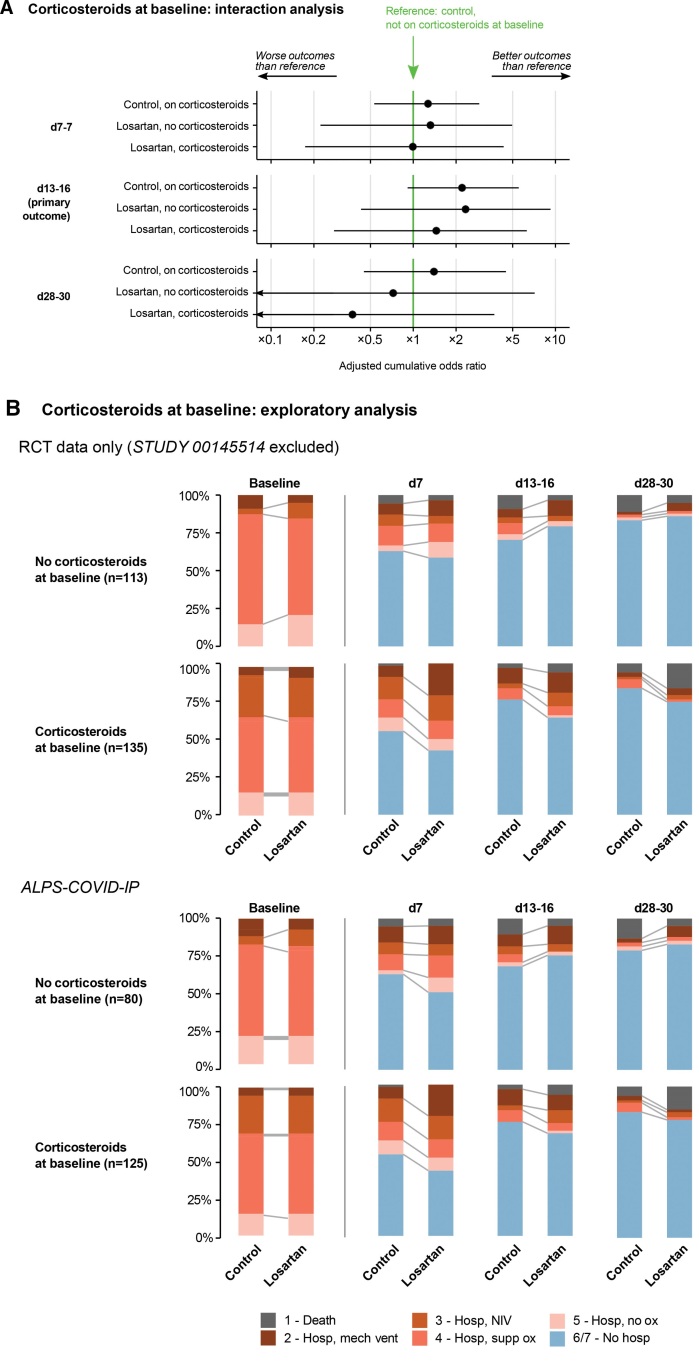
Corticosteroid interaction analyses. (A) Interaction coefficients comparing outcomes for individuals taking corticosteroids at baseline and/or assigned to losartan with outcomes for those not taking corticosteroids at baseline and assigned to control. Estimates are posterior means with 95% credible intervals. (B) Ordinal outcome data stratified by corticosteroids at baseline. Data are shown for all included RCTs and separately for ALPS-COVID IP, the largest included RCT.

In our interaction analysis, we found evidence that losartan had a worse effect for those taking corticosteroids at baseline compared with those who were not after adjusting for other baseline covariates (ratio of adjusted ORs 0.29, 95% CrI 0.08–0.99). Despite this, our model does not confidently predict an effect of losartan either when taking or not taking baseline corticosteroids, after adjusting for other covariates and with other interaction covariates set at their reference values (estimated adjusted OR without baseline steroids 2.24, 95% CrI 0.39–9.26; for a reference individual baseline steroids 0.65, 95% CrI 0.10–3.22); reference values are age 55, a baseline ordinal score of 5, symptom onset 7 days before enrollment, and no baseline comorbidities. Only ALPS-COVID IP had substantial numbers of patients both taking and not taking corticosteroids at baseline. The interaction was apparent in a post hoc exploratory analysis of both the raw outcome data from the 3 randomized trials, and the raw data from ALPS-COVID IP alone, though patients on corticosteroids at baseline also tended to be sicker as measured by the COVID ordinal score (see Figs. 3B and 4, and Table S7, Supplemental Digital Content, http://links.lww.com/MD/J48, which shows conditional covariate effects and between-study heterogeneity).

After adjusting for differences in the distribution of measured covariates, we did not find appreciable heterogeneity in treatment effect estimates across studies, either in the main model or the model with treatment-covariate interactions (main model: posterior mean of *τ* on the log-odds scale 0.98, 95% CrI 0.05–3.32; model with treatment-covariate interactions: posterior mean of *τ* 0.65, 95% CrI 0.02–2.57) (see Fig. 3B).

### 3.6. Secondary and safety outcomes

Ordinal outcome scores were similar between losartan and control patients in the pooled population at day 7 (model-standardized OR 0.87, 95% CrI 0.63–1.22; plug-in OR 0.81, 95% CI 0.53–1.21), and between days 28–30 (model-standardized OR 0.92, 95% CrI 0.52–1.61; plug-in OR 0.82, 95% CI 0.44–1.49). Adjusted analyses also yielded no convincing evidence at these time-points (day 7 conditional OR 0.99, 95% CrI 0.16–3.53; day 28–30 conditional OR 0.76, 95% CrI 0.02–3.32).

Mortality was also similar between losartan and control patients. In the pooled population, 9 of 169 control patients had died by day 13-16 compared with 6 of 156 patients assigned losartan (model-standardized risk difference 0.00, 95% CrI −0.02 to 0.03; plug-in risk difference 0.01, 95% CI −0.04 to 0.07), and 13 of 169 control patients died by day 28–30 compared with 14 of 156 losartan patients (model-standardized risk difference −0.01, 95% CrI −0.05 to 0.04; plug-in risk difference -0.01, 95% CI -0.08 to 0.05). Results were similar when excluding patients from STUDY 00145514 (Table 2). In a baseline risk subgroup analysis, our model confidently predicts only small effects of losartan on mortality on the risk difference scale for patients predicted to be at low risk of death under control (see Fig. 3C and Figure S1, Supplemental Digital Content, http://links.lww.com/MD/J49, which illustrates risk differences in day 13–16 mortality rate by subgroup).

There were similar rates of mechanical ventilation between enrollment and day 28 (21% [n = 32] losartan vs 16% [n = 27] control; these percentages exclude missing values). Losartan and control patients had a median post-enrollment length of stay of 7 and 6 days, respectively. Results were similar when including only RCT patients and excluding patients from STUDY 00145514.

When considering only RCTs, losartan patients had numerically higher AE and SAE rates per patient overall (AEs 0.63 [n = 79] vs 0.54 [n = 66] per patient; SAEs 0.55 [n = 69] vs 0.35 [n = 43] per patient). These differences were partly due to differences in hypotension AEs (0.11 [n = 14] vs 0.07 [n = 9] per patient) and hypotension SAEs (0.13 [n = 17] vs 0.05 [n = 6] events per patient). See Table 2 for further details.

### 3.7. Model checking and sensitivity analyses

Results were qualitatively similar using models fit only to data from RCTs (Fig. 2 and Table 2). Posterior predictive checks indicated good marginal within-sample fit of the models (see Figure S2, Supplemental Digital Content, http://links.lww.com/MD/J50, which illustrates posterior predictive check of day 13–16 ordinal score by site). For each timepoint considered (day 7, days 13–16, days 28–30), the main effects model had better estimated out-of-sample predictive performance than the model with treatment-covariate interactions (see Table S8, Supplemental Digital Content, http://links.lww.com/MD/J51, which tabulates estimated predictive performance of the models).

## 4. Discussion

Our IPD meta-analysis of 4 US-based trials in 325 COVID-19 inpatients comparing initiating losartan with control treatment found no evident benefit of losartan and is consistent with results of the 3 included RCTs considered individually.^[17–19]^ Neither the primary efficacy measurement (COVID-19 ordinal score at 13–16 days) nor our secondary outcomes (including day 13–16 and day 28–30 mortality) demonstrated improvement with losartan in the pooled study population. Overall AE and SAE rates were numerically higher with losartan, including higher hypotension AE and SAE rates. To our knowledge, this is the first published IPD meta-analysis of an ARB medication in COVID-19.

We observed no substantial differences in treatment effect between subgroups. However, we did find evidence suggesting that after adjusting for baseline covariates, losartan had worse effects for those taking corticosteroids at baseline. We were motivated to investigate this potential interaction by results from a recent trial of convalescent plasma in hospitalized COVID-19 patients requiring noninvasive supplemental oxygen^[30]^; investigators found an uncertain benefit of convalescent plasma overall, but evidence of a potential benefit in those patients not taking remdesivir and corticosteroids at randomization. There are several possible explanations of our finding. Though we are not aware of any direct pharmacokinetic or pharmacodynamic interactions between losartan and corticosteroids, corticosteroid use at enrollment was associated with more severe disease. Our interaction model included only a linear term for the numeric baseline ordinal score, so it is possible that the losartan-corticosteroid interaction reflects a nonlinear interaction between losartan and extreme levels of the baseline ordinal scale, and the sickest patients may be more likely to have complications such as hypotension, sepsis, AKI, or electrolyte abnormalities. The observed association also may be explained by changing standard of care over time associated with corticosteroid use, measuring the association for corticosteroid use at baseline rather than at any time during hospitalization, ecological biases, other unmeasured factors, or by chance.

Our equivocal efficacy findings and adverse safety signals are broadly consistent with those of ALPS-COVID IP, the largest study included in our meta-analysis. Our finding of higher hypotension rates is consistent with ALPS-COVID IP’s finding of fewer vasopressor-free days with losartan. ALPS-COVID IP and COVID MED are included in a separate International Society for Hypertension-led aggregate data meta-analysis of ACEi/ARB COVID-19 trials.^[31]^ In line with our results, that meta-analysis showed no mortality benefit, yet it differed from our results in that it found higher AKI rates—but not hypotension requiring inotropes rates—with ACEi/ARB medications.

Limitations of our study include: a relatively small sample size; inclusion of both randomized and non-randomized trials with both masked and open-label designs, as well as varying treatment arm dosing regimens and controls; inclusion of trials with enrollment at different pandemic stages and thus potential differences in standard of care; risk of bias from the studies’ randomization processes, deviations from protocol, missingness and imputation requirements, and outcome measurements; a limited dataset due to exclusion of 2 unpublished US/Canada-based studies for which principal investigators declined participation, as well as trials outside the US/Canada; and imperfect pre-specification due to SAP modifications after data harmonization and the inclusion of already-published results. Although trials studying any ACEi/ARB were targeted, all studies included in our analysis evaluated the ARB losartan, so our findings cannot be generalized to other ARB or ACEi medications.

## 5. Conclusions

Our IPD meta-analysis of 4 trials from the United States and Canada that evaluated *de novo* prescription of losartan medications for the treatment of COVID-19 in hospitalized patients found equivocal evidence for benefit in the overall pooled population and in subgroups. We also observed hypotension safety signals and a potential treatment interaction for those patients on corticosteroids at baseline; losartan appeared to have worse effects for that group. Our study, though limited by its small sample size, does not support the use of losartan to treat newly hospitalized COVID-19 patients outside of clinical trials, particularly for patients also taking corticosteroids.

## Acknowledgments

We are grateful for the efforts of our nonauthor collaborators in the Pandemic Response COVID-19 Research Collaboration Platform for ACEi/ARB Pooled Analyses, including investigators and support staff for the trials analyzed (see Appendix S1, Supplemental Digital Content, http://links.lww.com/MD/J52, which lists study collaborators). We thank Megan R. Clark and Emily Bartlett of Johns Hopkins University for their assistance preparing the manuscript. We appreciate the contributions made by members of the Trial Innovation Network and the COVID-19 Collaboration Platform. This collaboration is based on research using data from ALPS-COVID IP, COVID ARB, COVID MED, and STUDY 00145514 that has been made available through Vivli, Inc. Vivli has not contributed to or approved, and is not in any way responsible for, the contents of this publication.

## Author contributions

**Conceptualization:** Leon Di Stefano, Daniel O. Scharfstein, Barbara E. Bierer, Daniel F. Hanley, Daniel Freilich.

**Data curation:** Leon Di Stefano, Malathi Ram.

**Formal analysis:** Leon Di Stefano, Daniel O. Scharfstein, Tianjing LI, Sheriza N. Baksh, Daniel Freilich.

**Funding acquisition:** Daniel F. Hanley.

**Investigation:** Leon Di Stefano, Malathi Ram, Daniel O. Scharfstein, Tianjing LI, Preeti Khanal, Sheriza N. Baksh, Nichol McBee, Charles D. Bengtson, Anne Gadomski, Matthew Geriak, Michael A. Puskarich, Matthias A. Salathe, Aletta E. Schutte, Christopher J. Tignanelli, Jennifer Victory, Barbara E. Bierer, Daniel F. Hanley, Daniel Freilich.

**Methodology:** Leon Di Stefano, Daniel O. Scharfstein, Tianjing LI, Sheriza N. Baksh, Charles D. Bengtson, Anne Gadomski, Matthew Geriak, Michael A. Puskarich, Matthias A. Salathe, Aletta E. Schutte, Christopher J. Tignanelli, Barbara E. Bierer, Daniel F. Hanley, Daniel Freilich.

**Project administration:** Malathi Ram, Preeti Khanal, Nichol McBee, Jennifer Victory, Daniel F. Hanley, Daniel Freilich.

**Resources:** Daniel F. Hanley.

**Software:** Leon Di Stefano.

**Supervision:** Daniel O. Scharfstein, Nichol McBee, Daniel F. Hanley, Daniel Freilich.

**Validation:** Leon Di Stefano.

**Visualization:** Leon Di Stefano, Malathi Ram, Preeti Khanal.

**Writing – original draft:** Leon Di Stefano, Daniel Freilich.

**Writing – review & editing:** Leon Di Stefano, Daniel O. Scharfstein, Tianjing LI, Sheriza N. Baksh, Charles D. Bengtson, Anne Gadomski, Matthew Geriak, Michael A. Puskarich, Matthias A. Salathe, Aletta E. Schutte, Christopher J. Tignanelli, Barbara E. Bierer, Daniel F. Hanley, Daniel Freilich.

## Supplementary Material


























