# Supplementary material for: Losartan in hospitalized patients with COVID-19 in North America: An individual participant data meta-analysis
Source: Medicine (Baltimore). 2023 Jun 9;102(23):e33904. doi: 10.1097/MD.0000000000033904 (PMC10256351; doi:10.1097/MD.0000000000033904)
Supplement: Supplementary file 1 [file medi-102-e33904-s001.pdf]

Table S1. Data Dictionary from Data Harmonization Spreadsheet

In addition to the variables below, we requested inclusion and exclusion criteria from each trial.

Variable	Format	Description and comments
Patient ID	(Study-specific format)	
Other ID(s)	(Study-specific format)	
Treatment group	(Study-specific format)	
Enrollment date (year)	yyyy	Year of enrollment
Symptom onset date	numeric (days)	Enrollment date - symptom onset date
Screening date	numeric (days)	Enrollment date - screening date
Admission date	numeric (days)	Enrollment date - admission date
Randomization date	numeric (days)	Randomization date - enrollment date
Date of first dose	numeric (days)	Date of first dose - date of enrollment (numeric; days)
Date of last dose	numeric (days)	Date of last dose - date of enrollment
Ordinal outcome scale	Ordinal scale:<br>1 = Death<br>2 = Hospitalized, on invasive mechanical ventilation or extracorporeal membrane oxygenation (ECMO)<br>3 = Hospitalized, on non-invasive ventilation or high flow oxygen devices<br>4 = Hospitalized, requiring	Baseline ordinal scales as well as all available through day 40 post enrollment (Day 0, Day 7, Day 14, Day 28/30 are most important)

Variable	Format	Description and comments
	supplemental oxygen<br>5 = Hospitalized, not requiring supplemental oxygen<br>6 = Not hospitalized, limitation on activities<br>7 = Not hospitalized, no limitations on activities	
Date at which ordinal scale is measured	numeric (days)	Date of ordinal outcome status – date of enrollment
Hospitalization length of stay	numeric (days)	Duration in days between enrollment date and day 28 post enrollment
Duration of mechanical ventilation	numeric (days)	Duration in days between enrollment date and day 28 post enrollment
Number of SAEs	numeric (count)	Between enrollment and day 28
Number of AEs	numeric (count)	Between enrollment and day 28
All-cause mortality	1 = Yes, 0 = No	Between enrollment and day 28
All-cause mortality – date of death	numeric (days)	Between enrollment and day 28
AKI SAEs	numeric (count)	Between enrollment and day 28
AKI AEs	numeric (count)	Between enrollment and day 28
Hyperkalemia SAEs	numeric (count)	Between enrollment and day 28
Hyperkalemia AEs	numeric (count)	Between enrollment and day 28
Hypotension SAEs	numeric (count)	Between enrollment and day 28

Variable	Format	Description and comments
Hypotension AEs	numeric (count)	Between enrollment and day 28
Age (truncated at ≥90)	numeric (years)	Age rounded *down* to beginning of 5-year bracket – e.g. 43 → 40; 39 → 35 — truncated at ≥90 years of age
Sex	1 = Male, 2 = Female	
Race	1 = American Indian/Alaska Native;<br>2 = Asian;<br>3 = Black/African American;<br>4 = Native Hawaiian/Pacific Islander;<br>5 = White;<br>6 = Multiple;<br>7 = Other/declined;<br>8 = Unknown/unavailable	
Ethnicity	0 = Not of Hispanic, Latinx, or Spanish origin<br>1 = Hispanic, Latinx, or Spanish origin<br>2 = Unknown	
BMI	numeric	
On mechanical ventilation at enrollment	1 = Yes, 0 = No	
AIDS (do not include HIV-positive without AIDS criteria)	1 = Yes, 0 = No	
Cerebrovascular disease	1 = Yes, 0 = No	

Variable	Format	Description and comments
A prior myocardial infarction	1 = Yes, 0 = No	
Congestive heart failure	1 = Yes, 0 = No	
Dementia	1 = Yes, 0 = No	
COPD	1 = Yes, 0 = No	
Asthma	1 = Yes, 0 = No	
History of hypertension	1 = Yes, 0 = No	
HIV positive (without AIDS)	1 = Yes, 0 = No	
Solid tumor	1 = Yes, 0 = No	
Liver disease	1 = Yes, 0 = No	
Diabetes mellitus	1 = Yes, 0 = No	
Cigarette or tobacco smoking	1 = Current or ever, 0 = Never	
Vaping	1 = Current or ever, 0 = Never	
Charlson comorbidity index	numeric	A precomputed Charlson comorbidity index, as in Charlson et al (1987)
Concurrent corticosteroid use at any point from enrollment through 28 days post-first dose	1 = Yes, 0 = No	
