# Supplementary material for: Losartan in hospitalized patients with COVID-19 in North America: An individual participant data meta-analysis
Source: Medicine (Baltimore). 2023 Jun 9;102(23):e33904. doi: 10.1097/MD.0000000000033904 (PMC10256351; doi:10.1097/MD.0000000000033904)
Supplement: Supplementary file 2 [file medi-102-e33904-s002.pdf]

Table S2. Changes to the Prespecified Statistical Analysis Plan

Time of change	Category of change	Change to SAP	Reason for change
After examining baseline data and outcome missingness	Outcome and estimand definitions	Change primary outcome definition to d13-16 score	Many imputed values/non-direct follow-up at d28-30
		Simplified secondary outcomes	Simplicity of analysis
		Use regression coefficients rather than relative risks for conditional interaction effects	Simplicity of analysis
		Change standardization estimand to geometric mean of the cumulative odds ratios	Reduce analysis runtime; simplicity
	Statistical model	Main model has no treatment interactions apart from treatment-by-study	Simplicity of analysis; anticipated model fit; small sample size
		Use default priors from the “brms” package	Simplicity of analysis
		Smaller number of adjustment variables; remove spline terms	Anticipated model fit; small sample size
		Smaller number of covariates for subgroup & interaction analyses	Simplicity of analysis; anticipated model fit; small sample size

		Simplified covariate coding; use tertiles in the pooled population for subgroup analyses	Simplicity of presentation
	Sensitivity analyses	Removed some sensitivity analyses	Simplicity of analysis
After analyzing outcome data (post-hoc)	Statistical model	Used quintiles rather than tertiles to bin baseline risk score values	Clarity of presentation
	Sensitivity analyses	Add sensitivity analyses for STUDY 00145514	Potential biases and effects on the pooled analysis of non-concurrent controls
