# Supplementary material for: Losartan in hospitalized patients with COVID-19 in North America: An individual participant data meta-analysis
Source: Medicine (Baltimore). 2023 Jun 9;102(23):e33904. doi: 10.1097/MD.0000000000033904 (PMC10256351; doi:10.1097/MD.0000000000033904)
Supplement: Supplementary file 3 [file medi-102-e33904-s003.pdf]

Losartan in Hospitalized Patients with Covid-19 in North America:
An Individual Patient Data Meta-Analysis

Supplemental Methods

1. Primary outcome model

1.1 Model specification

We fit a Bayesian proportional odds ordinal regression model for the ordinal outcome score measured at day 13-16. For individuals with multiple measurements in this time interval, the outcome was taken as the earliest measurement.

The model included indicators for treatment and study, as well as treatment-by-study interactions. We adjusted for the following individual-level covariates: sex, age, number of baseline comorbidities, baseline corticosteroid use, symptom onset days before enrollment, and baseline COVID outcome scale. Our primary analysis model did not include treatment-covariate interactions.

Model description. Let individual patients be indexed by *i*. Each patient has a vector of baseline covariates X_i and is assigned to treatment T_i, either losartan ($T_i = 1$) or control/standard of care/placebo ($T_i = 0$). Let the primary outcome for individual *i* be denoted Y_i with levels indexed by *l*. Finally, let $Y_i(0)$ and $Y_i(1)$ be the potential outcomes for individual *i* under control and treatment respectively, regardless of treatment actually received.

The proportional odds model takes the form:

$$\text{logit}\, P[Y_i \leq l \mid X_i, T_i] = \theta_l - \eta_i;\ l = 1, \ldots, 5$$

where $\theta_1, .., \theta_5$ are cutpoints that are common to all individuals and the linear predictor takes the form

$$\eta_i = T_i\delta + X_i^{\top}\beta + \alpha_{0,study} + T_i\,\alpha_{1,study}$$

- The X_i vector contains the predictors
  - age,

- ◦ sex,
- ◦ baseline ordinal score (as a factor),
- ◦ number of baseline comorbidities (truncated at 4),
- ◦ baseline corticosteroid use, and
- ◦ symptom onset days before enrollment.

- The coefficients δ and β are given uniform priors.
- The intercepts θ_l are modeled with a Student-*t* prior with degrees of freedom 3 and scale parameter 2.5, subject to the constraint that $\theta_1 \leq \cdots \leq \theta_5$.
- The coefficients $\alpha_{0,study}$ and $\alpha_{1,study}$ are modeled as independent mean-zero Normal random effects, each with their own standard deviation parameter independently distributed as a half Student-*t* distribution with 3 degrees of freedom and scale parameter 2.5. The standard deviation of $\alpha_{1,study}$ is denoted τ.

The model was fit using R, and the library "brms."

We computed three estimates of the overall effect of losartan:

1. A "plug-in" estimate of the marginal cumulative odds ratio, taken from a proportional odds model fit by maximum likelihood with only the treatment indicator as a predictor.
2. A "model-standardized" estimate of the marginal cumulative odds ratio. We produced this by drawing from the posterior distribution of predicted probabilities of the potential outcomes in the pooled study population, averaging these across patients for each outcome level under each treatment, and using these averages to compute the effect measure of interest, which we took as the geometric mean of cumulative odds ratios over the six cut-points of the ordinal outcome scale. (This is equal to the cumulative odds ratio when the proportional odds assumption holds.) This model-standardized estimate is our primary estimate of treatment efficacy.
3. An estimate of the conditional (adjusted) cumulative odds ratio; this is δ in the model above.

1.2 Subgroup and interaction effects

In the model for investigating interactions and subgroup effects, we added individual-level interaction terms so that the linear predictor takes the form:

$$\eta_i = T_i\delta + X_i^{\top}\beta + T_i Z_i^{\top}\gamma + \alpha_{0,study} + T_i\ \alpha_{1,study}$$

The model is as in the main analysis except that

- The Z_i vector contains the predictors
  - age,
  - baseline ordinal score,
  - baseline corticosteroid use, and
  - symptom onset days before enrollment.
- The Z_i terms and corresponding terms in X are coded as in the table below.
- The γ term was given a uniform prior.

For each covariate, we computed three kinds of effect estimates, as in the overall analysis:

1. "Plug-in" estimates of subgroup cumulative odds ratios, taken from a proportional odds model fit by maximum likelihood within the subgroup of interest.
2. "Model-standardized" estimates of subgroup cumulative odds ratios, computed as in the overall analysis. (Defined as the mean of the log cumulative odds ratios across cut-points when the proportional odds assumption does not hold.)
3. Estimates of the interaction coefficients γ from the model above.

The following table describes the coding of interaction terms in the model and the covariate bins used to define subgroups for continuous covariates:

Covariate	Coding	Reference level	Subgroups
age	(age in years – median age)/10	median age	split by tertiles in the pooled population, rounded to the nearest 5y

baseline ordinal score	5 – the numeric score	5 - hospitalized, not requiring supplemental oxygen (the highest possible value for inpatients)	(all levels)
baseline corticosteroids	1 = corticosteroids<br>0 = no corticosteroids	0 = no corticosteroids	(both levels)
symptom onset days before enrollment	(days – median days)/(7 days)	median value in the data	split by tertiles in the pooled population, rounded to the nearest day

We also conducted a subgroup analysis based on a "baseline risk score." This score is given by each individual's expected linear predictor under the control arm (marginalized over study effects); subgroups were formed using quintiles in the pooled population.

2. Secondary outcome analyses

The ordinal score secondary outcomes (at day 7 and at day 28-30) were analyzed in the same manner as the primary outcome.

For the mortality secondary outcomes (at day 13-16 and at day 28-30), we computed plug-in and model-standardized estimates of risk differences using the same models as for the corresponding ordinal score outcome in the standardization.

3. Model checking and robustness

For the primary outcome model, we conducted posterior predictive checks and computed cross validation-based estimates of out-of-sample predictive accuracy.

4. Missing data

We assessed the extent of missing baseline covariate data at the design stage. If the missingness was judged to be minor and sporadic, the missing values were filled in using multiple imputation based on a selection of other baseline covariates, but excluding treatment assignment and

outcome. If the missingness was judged to be severe or systematic, the corresponding baseline variables were followed up with the individual study investigators and, if necessary, excluded from the analysis.

Missing outcome data was modeled under the assumption that it was missing at random conditional on the covariates included in our regression model.
