# Supplementary material for: Losartan in hospitalized patients with COVID-19 in North America: An individual participant data meta-analysis
Source: Medicine (Baltimore). 2023 Jun 9;102(23):e33904. doi: 10.1097/MD.0000000000033904 (PMC10256351; doi:10.1097/MD.0000000000033904)
Supplement: Supplementary file 5 [file medi-102-e33904-s005.pdf]

Table S3. Trial Characteristics: Primary, Secondary, and Safety Outcomes

Trial	Primary outcome	Secondary outcomes	Safety outcomes
ALPS-COVID IP (NCT04312009)	Difference in ratio of arterial partial pressure of oxygen to fraction of inspired oxygen (PaO2:FiO2) at 7 days	• Safety and tolerance of losartan, as assessed by:<br>• Mean daily hypotensive episodes through day 10<br>• Use of vasopressors or fluid bolus for hypotension through day 10<br>• New or worsening renal failure through day 10<br>• Sequential Organ Failure Assessment (SOFA) score through day 10<br>• Change in estimated PaO2:FiO2 ratio daily between groups<br>• In-hospital, 28-day, and 90-day mortality<br>• Resource utilization, as assessed by:<br>• Mean ICU admissions through day 10<br>• Mean ventilator-free days through day 10<br>• Mean therapeutic oxygen-free days through day 10<br>• Mean vasopressor-free days through day 10<br>• Mean length of ICU and hospital stays through day 10<br>• Incidence of respiratory failure through day 10 (requiring BiPAP, high-flow nasal cannula, mechanical ventilation, or extracorporeal membranous oxygenation [ECMO])<br>• Change in PROMIS dyspnea scale and SF-12 scores from baseline to day 10<br>• Disease severity rating at day 15	• AEs and SAEs through day 15<br>• SAEs of interest (continuous monitoring and reporting): deaths, cardiovascular events (need for vasoactive drugs or fluids for hypotension), and respiratory events (worsening hypoxia, worsening acute respiratory distress syndrome, or new respiratory failure)

Trial	Primary outcome	Secondary outcomes	Safety outcomes
		• Change in viral load (nasopharyngeal or oropharyngeal swab and blood)	
STUDY 00145514<br><br>(NCT04335123)	Number of participants with treatment-related adverse events at day 14	• Number of days on supplemental oxygen due to COVID-19 through day 14<br><br>• Incidence of mechanical ventilation use through day 14<br><br>• Days on mechanical ventilation through day 14<br><br>• Incidence of non-invasive positive pressure ventilation or heated high-flow nasal cannula use through day 14<br><br>• Days on non-invasive positive pressure ventilation or high flow nasal cannula through day 14<br><br>• Incidence of transfer to ICU from non-ICU hospital bed through day 14<br><br>• ICU length of stay (days) through day 14<br><br>• In-hospital mortality rate<br><br>• Hospital length of stay (days) through day 14<br><br>• Cumulative incidence of SAEs through day 14<br><br>• Cumulative incidence of AEs through day 14<br><br>• Change in oxygenation from baseline to day 14<br><br>• Incidence of medications with possible antiviral activity (hydroxychloroquine, lopinavir/ritonavir, ribavirin or remdesivir) or adjunctive therapy use (e.g., tocilizumab) through day 14<br><br>• Incidence (and length in days) of extracorporeal membrane oxygenation use through day 14<br><br>• Incidence (and length in days) of renal replacement therapy use through day 14	• AEs and SAEs through day 14 (or study completion, if sooner)

Trial	Primary outcome	Secondary outcomes	Safety outcomes
		• Intolerance of high-dose (50 mg) losartan after tolerating 25 mg through day 14<br>• Change in SARS-CoV-2 viral load determined by quantitative PCR	
COVID ARB<br>(NCT04340557)	Number of participants requiring mechanical ventilation	• Incidence of transfer to ICU from non-ICU hospital bed<br>• Mean use of oxygen therapy (in liters of oxygen)	• Length of hospital stay<br>• In-hospital mortality
COVID MED<br>(NCT04328012)	National Institute of Allergy and Infectious Diseases COVID-19 Ordinal Severity Scale	• Hospital length of stay at 60 days<br>• ICU length of stay at 60 days<br>• Mechanical ventilation use at 60 days<br>• Survival at 60 days	• Complications<br>• AEs/SAEs
