# Supplementary material for: Losartan in hospitalized patients with COVID-19 in North America: An individual participant data meta-analysis
Source: Medicine (Baltimore). 2023 Jun 9;102(23):e33904. doi: 10.1097/MD.0000000000033904 (PMC10256351; doi:10.1097/MD.0000000000033904)
Supplement: Supplementary file 6 [file medi-102-e33904-s006.pdf]

Table S4. Trial Characteristics: Treatment Groups, Participant Follow-Up, and Inclusion/Exclusion Criteria

Trial overview	Study arm dose	Follow-up	Inclusion criteria	Exclusion criteria
**ALPS-COVID IP**<br>(NCT04312009)<br><br>• Sponsor: University of Minnesota<br>• Blinded<br>• Sample size: 200 planned; 205 actual<br>• Participant age: ≥18 years<br>• Study arms: 2<br>• Length of treatment: 10 days or until hospital discharge	**Losartan:** 50 mg twice daily<br><br>**Control:** oral placebo (microcrystalline methylcellulose, gelatin capsule)	90 days (daily assessments through day 10 or discharge, whichever comes first; follow-up on days 15, 28, and 90)	• Presumptive positive laboratory test for Covid-19 based on local laboratory standard<br>• Age ≥18 years of age<br>• At least one coronavirus symptom per CDC symptom list (https://www.cdc.gov/coronavirus/2019-ncov/symptoms-testing/symptoms.html)<br>• Admission to the hospital with a respiratory SOFA ≥1 and increased oxygen requirement compared to baseline among those on home O2<br>• Randomization within 48 hours of presentation of hospital admission or within 48 hours of a positive test result, whichever is later	• Randomization >48 hours of admission order or positive test result, whichever is later<br>• Randomization >48 hours of admission order or positive test result, whichever is later<br>• Currently taking an angiotensin converting enzyme inhibitor (ACEi) or angiotensin receptor blocker (ARB)<br>• Prior reaction or intolerance to an ARB or ACE inhibitor, including but not limited to angioedema<br>• Pregnant or breastfeeding<br>• Lack of negative urine or serum pregnancy test<br>• Not currently taking a protocol-allowed version of contraception: intrauterine device, Depo-formulation of hormonal contraception (e.g. medroxyprogesterone acetate/Depo-Provera), subcutaneous contraceptive (e.g. Nexplanon), daily oral contraceptives with verbalized commitment to taking daily throughout the study period; use of condoms or agreement to abstain from sexual intercourse during the study. All women of childbearing age enrolled in this fashion will be informed of the teratogenic risks. If enrolled by a legally authorized representative, patient will be informed of the risks after regaining capacity.<br>• Patient-reported history or electronic medical record history of kidney disease, defined as:

Trial overview	Study arm dose	Follow-up	Inclusion criteria	Exclusion criteria
				a. Any history of dialysis<br>b. History of chronic kidney disease stage IV<br>c. Estimated Glomerular Filtration Rate (eGFR) of <30ml/min/1.73 m2 at time of randomization<br>• Severe dehydration at enrollment in the opinion of the investigator or bedside clinician<br>• Most recent mean arterial blood pressure prior to enrollment <65 mmHg<br>• Patient-reported history or electronic medical record history of severe liver disease, defined as:<br>a. Cirrhosis<br>b. History of hepatitis B or C<br>c. Documented AST or ALT >3 times the upper limit of normal measured within 24 hours prior to randomization<br>• Potassium >5.0 within 24 hours prior to randomization unless a repeat value was ≤5.0<br>• Treatment with aliskiren<br>• Inability to obtain informed consent from participant or legally authorized representative<br>• Enrollment in another blinded randomized clinical trial for COVID
**STUDY 00145514**<br>(NCT04335123)	**Losartan:** 25 mg once daily on days 0-2; 50 mg once daily	Up to 14 days (daily assessments on days 0	• Age ≥18 years admitted to the University of Kansas Health System	• Pregnancy<br>• Respiratory failure due to a process other than COVID-19

Trial overview	Study arm dose	Follow-up	Inclusion criteria	Exclusion criteria
• Sponsor: University of Kansas<br>• Open label<br>• Sample size: 34 planned; 77 actual (including post-hoc external controls)<br>• Participant age: ≥18 years<br>• Study arms: 2<br>• Length of treatment: 14 days or until resolution of respiratory failure, hospital discharge, or protocol-defined stoppage criteria is met (whichever comes first)	from day 3 until study completion<br><br>**Control:** standard care (post-hoc external historical)	through study completion day)	• Confirmation of SARS-CoV-2 infection by PCR testing<br>• Hypoxic respiratory failure requiring mechanical ventilation or oxygen OR a SpO2 ≤94% on room air or a PaO2/FiO2 ratio <300 OR tachypnea (respiratory rate ≥24 breaths/min)<br>• Other concomitant medications such as antivirals and hydroxychloroquine are allowed<br>• Participants prescribed standard of care (SOC) losartan (25 mg once daily) within 48 hours of consenting may be considered for enrollment if eligibility criteria are met based on EMR data assessment (i.e. no other ARB or ACE prior to SOC medication administration). If participant is eligible and signs consent form, investigational losartan at 25 mg once daily will be ordered to replace the SOC prescription on the following scheduled dose.	• Intolerance to ARBs<br>• Previous treatment with an ARB or ACE inhibitor (see exception in inclusion criteria)<br>• Current chronic use of medication with known interactions with losartan including NSAIDs (intermittent prior use is acceptable), potassium supplementation, or aliskiren<br>• Blood pressure less than 90 mm Hg systolic or 60 mm Hg diastolic recorded on at least two readings 30 min apart<br>• Need for vasopressors, unless norepinephrine ≤0.1 μg/kg/min<br>• Hyperkalemia (serum K+ >5.5 mM)<br>• Known cardiac failure (left ventricular ejection fraction ≤35%), renal insufficiency (Cockcroft-Gault <30 mL/min/1.73 m2 or urinary output <20 mL/h), hepatic failure (LFTs >5x normal upper limit)<br>• Known renal artery stenosis<br>• Neurological, psychiatric, endocrine, or neoplastic diseases that are judged to interfere with participation<br>• Participating in another interventional trial (including one for COVID-19) that excludes participation<br>• Meeting all inclusion criteria for more than 48 hours prior to day 0
**COVID ARB**	**Losartan:** Standard care plus 12.5 mg	10 days	• Confirmed COVID-19 positive test result	• Severe allergy to any ARB or ACE-inhibitor, including angioedema

Trial overview	Study arm dose	Follow-up	Inclusion criteria	Exclusion criteria
(NCT04340557)<br><br>• Sponsor: Sharp Healthcare<br>• Open label<br>• Sample size: 200 planned; 31 actual<br>• Participant age: ≥18 years<br>• Study arms: 2<br>• Length of treatment: 10 days	twice daily; can be increased on days 2-10 if tolerated<br><br>**Control:** Standard care		• Mild to moderate respiratory symptoms of COVID-19<br>• Systolic blood pressure ≥100 mm Hg<br>• Screen within 3 days of a positive COVID-19 test<br>• Age ≥18 years old<br>• Access to a phone or other electronic device capable of receiving phone or video calls and email for remote consent<br>• Able to read, write, and speak English or Spanish fluently<br>• Capacity to provide consent or an appropriate LAR to provide consent<br>• Negative pregnancy test for women of childbearing potential if randomized to the study treatment arm	• In the intensive care unit at screening<br>• Home meds include any kind of ACE inhibitor or ARB<br>• Acute kidney injury (50% reduction in GFR from baseline at admission to any time during treatment in the study treatment arm)<br>• Hyperkalemia >5.0 mmol/L at baseline or any time during treatment in the study treatment arm<br>• Creatinine clearance <30 ml/min at baseline or any time during treatment in the study treatment arm
**COVID MED**<br>(NCT04328012)<br><br>• Sponsor: Bassett Healthcare<br>• Blinded<br>• Sample size: up to 4,000 planned; 14 actual<br>• Participant age: ≥18 years	**Arm 1**: standard care plus lopinavir/ritonavir 400 mg/100 mg twice daily for up to 14 days<br><br>**Arm 2**: standard care plus hydroxy-chloroquine: 400 mg twice daily on day 1; 200 mg twice daily on days 2-14	60 days (assessments at baseline and on days 1-7, day 14, day 30, and day 60)	• Hospitalized patient<br>• Age ≥18 years<br>• Able to ingest oral medication or be administered medication via gastric tube or equivalent<br>• Laboratory confirmation of SARS-CoV-2 infection within 1 week prior to randomization<br>• Randomization within 72 hours of hospital admission<br>• Negative pregnancy test for reproductive age women	General (all groups) exclusions:<br>• End stage renal disease (ESRD) NOT undergoing renal replacement therapy<br>• Severe hepatic insufficiency (LFTs >5 times the upper limit of normal or known ESLD or cirrhosis)<br>• Nausea/vomiting or aspiration risk precluding oral medications unless can be given by gastric tube<br>• Use of another SARS-CoV-2 directed medication empirically or within another clinical trial within prior week

Trial overview	Study arm dose	Follow-up	Inclusion criteria	Exclusion criteria
• Study arms: 4<br>• Length of treatment: Up to 14 days	**Arm 3**: standard care plus losartan 25 mg once daily for up to 14 days; placebo (Tic Tacs in blank capsules) once daily to replicate and control for twice-daily dosing in other arms<br><br>**Arm 4**: standard care plus placebo (Tic Tacs in blank capsules) twice daily for up to 14 days		• Patient or legally authorized representative able to provide informed consent	• Pregnancy or breast feeding<br>• Absence of dependable contraception in reproductive age women<br>• Inability to provide or declined informed consent<br>Losartan group exclusions:<br>• Allergy or intolerance to losartan or other ARBs<br>• Already taking ACE or ARB (within 1 month)<br>• Hypotension at time of enrollment (SBP <100 mm Hg)<br>• Hyperkalemia (K ≥5.0 at time of screening or history of hyperkalemia)<br>• Severe renal dysfunction (estimated GFR <30 ml/min at time of screening or history of advanced renal disease)<br>• Severe volume depletion or acute kidney injury at time of enrollment<br>• Known cirrhotic ascites<br>• Known severe aortic or mitral valve stenosis<br>• Known unstented renal artery stenosis<br>• Co-administration with certain drugs due to CYP3A interactions if taken in <24 hours
