# Supplementary material for: Losartan in hospitalized patients with COVID-19 in North America: An individual participant data meta-analysis
Source: Medicine (Baltimore). 2023 Jun 9;102(23):e33904. doi: 10.1097/MD.0000000000033904 (PMC10256351; doi:10.1097/MD.0000000000033904)
Supplement: Supplementary file 7 [file medi-102-e33904-s007.pdf]

Table S5. Risk of Bias Assessment

Randomized trials†	Bias arising from the randomization process	Bias due to deviations from the intended intervention	Bias due to missing outcome data	Bias in the measurement of the outcome			Overall risk of bias
ALPS-COVID IP	Low	Low	Low	Low			**Low**
COVID ARB	Low	Low	Low	High			**High**
COVID MED	High	Low	Low	Low			**High**
Non-randomized studies‡	Bias due to confounding	Bias in selection of participants into the study	Bias in classification of interventions	Bias due to deviations from the intended intervention	Bias due to missing data	Bias in the measurement of the outcome	Overall risk of bias
STUDY 00145514	Moderate	Serious	Low	Low	Low	Low	**Serious**

†Assessed using Cochrane Risk of Bias 2 tool (RoB 2)

‡Assessed using the Risk of Bias In Non-randomized Studies of Intervention assessment tool (ROBINS-I)
