# Supplementary material for: Losartan in hospitalized patients with COVID-19 in North America: An individual participant data meta-analysis
Source: Medicine (Baltimore). 2023 Jun 9;102(23):e33904. doi: 10.1097/MD.0000000000033904 (PMC10256351; doi:10.1097/MD.0000000000033904)
Supplement: Supplementary file 8 [file medi-102-e33904-s008.pdf]

Table S6. Subgroup Effects for d13-16 ordinal score and mortality

	Standardized OR (95% CrI)	Plug-in OR (95% CI)	Standardized risk difference for mortality (95% CrI)	Plug-in risk difference for mortality (95% CI)
**Pooled population**	1.35 (0.62 to 2.62)	0.96 (0.59 to 1.54)	0.01 (-0.03 to 0.05)	0.01 (-0.04 to 0.07)
**Study**				
ALPS-COVID IP	1.09 (0.49 to 2.24)	0.90 (0.49 to 1.65)	0.01 (-0.05 to 0.05)	0.00 (-0.07 to 0.06)
STUDY 00145514	2.21 (0.97 to 4.70)	1.21 (0.47 to 3.30)	0.04 (0.00 to 0.08)	0.04 (-0.04 to 0.13)
COVID ARB	2.97 (0.86 to 10.75)	2.59 (0.43 to 21.08)	0.02 (0.00 to 0.07)	0.07 (-0.12 to 0.26)
COVID MED	0.86 (0.23 to 2.61)	NA	-0.02 (-0.15 to 0.12)	0.00 (NA)[a]
**Ordinal score at baseline**				
2: hosp, mech vent	1.65 (0.66 to 3.46)	1.68 (0.37 to 8.06)	0.08 (-0.08 to 0.22)	0.08 (-0.27 to 0.42)
3: hosp, NIV	1.01 (0.37 to 2.42)	0.71 (0.26 to 1.90)	0.00 (-0.11 to 0.11)	0.01 (-0.18 to 0.20)
4: hosp, supp ox	1.59 (0.65 to 4.17)	1.53 (0.71 to 3.39)	0.01 (-0.01 to 0.03)	0.03 (-0.03 to 0.09)
5: hosp, no ox	1.83 (0.73 to 4.75)	1.64 (0.43 to 8.02)	0.01 (0.00 to 0.02)	0.00 (NA)[a]
**Age (years)**				
20-49	1.32 (0.49 to 3.00)	0.72 (0.25 to 2.03)	0.01 (-0.02 to 0.04)	0.00 (NA)[a]
50-64	1.25 (0.58 to 2.61)	1.07 (0.51 to 2.28)	0.01 (-0.03 to 0.05)	-0.01 (-0.08 to 0.07)
65-90	1.44 (0.58 to 3.21)	0.88 (0.38 to 2.03)	0.02 (-0.05 to 0.10)	0.07 (-0.10 to 0.23)
**Baseline corticosteroids**				
No corticosteroids	2.07 (0.94 to 4.64)	1.45 (0.76 to 2.80)	0.03 (0.00 to 0.08)	0.04 (-0.03 to 0.10)
Corticosteroids	0.76 (0.28 to 1.86)	0.59 (0.28 to 1.21)	-0.01 (-0.08 to 0.04)	-0.01 (-0.10 to 0.07)
**Symptom onset days before enrollment**				
0-5	1.23 (0.56 to 2.72)	1.27 (0.55 to 3.06)	0.01 (-0.04 to 0.06)	0.02 (-0.10 to 0.15)
6-8	1.37 (0.59 to 2.73)	0.68 (0.27 to 1.63)	0.01 (-0.02 to 0.05)	0.00 (NA)[a]

9-28	1.40 (0.53 to 3.53)	1.37 (0.55 to 3.50)	0.02 (-0.03 to 0.07)	-0.02 (-0.12 to 0.08)
**Baseline risk group**				
1st	0.96 (0.24 to 3.10)	1.27 (0.26 to 6.90)	0.00 (-0.01 to 0.01)	0.00 (NA)[a]
2nd	1.29 (0.46 to 3.36)	2.01 (0.40 to 14.81)	0.00 (-0.01 to 0.02)	0.00 (NA)[a]
3rd	1.53 (0.60 to 4.10)	1.23 (0.33 to 5.14)	0.01 (-0.01 to 0.03)	0.03 (-0.05 to 0.11)
4th	1.45 (0.59 to 3.16)	1.22 (0.45 to 3.33)	0.01 (-0.04 to 0.06)	0.03 (-0.06 to 0.12)
5th	1.45 (0.60 to 3.25)	1.21 (0.50 to 2.93)	0.05 (-0.08 to 0.17)	0.09 (-0.14 to 0.32)

Model-standardized estimates are derived from the model with treatment-covariate interactions.

[a]There were no deaths in these subgroups.
