# Supplementary material for: Losartan in hospitalized patients with COVID-19 in North America: An individual participant data meta-analysis
Source: Medicine (Baltimore). 2023 Jun 9;102(23):e33904. doi: 10.1097/MD.0000000000033904 (PMC10256351; doi:10.1097/MD.0000000000033904)
Supplement: Supplementary file 9 [file medi-102-e33904-s009.pdf]

Table S7. Conditional Covariate Effects and Between-Study Heterogeneity

	**Odds ratio (95% CrI)**
Treatment effect for a reference individual on d13-16 ordinal score <br>*(age 55, a baseline ordinal score of 5, symptom onset 7 days before enrollment, no baseline corticosteroids, and no baseline comorbidities)*	2.24 (0.39 to 9.26)
Difference in treatment effect associated with …	**Ratio of odds ratios (95% CrI)**
1 point worse on the ordinal scale at baseline	0.87 (0.45 to 1.64)
10 years older	1.07 (0.74 to 1.55)
Baseline corticosteroids	0.29 (0.08 to 0.99)
Symptom onset 7 days earlier	1.35 (0.54 to 3.42)
Study-specific conditional treatment effects for a reference individual [covariate interactions model]	**Odds ratio (95% CrI)**
ALPS-COVID IP	2.22 (0.64 to 7.82)
STUDY 00145514	2.95 (0.84 to 11.32)
COVID ARB	2.93 (0.75 to 13.37)
COVID MED	2.33 (0.40 to 11.64)
	τ = 0.65 (on the log-odds scale; 95% CrI 0.02 – 2.57)
Study-specific conditional treatment effects [main effect only model]	**Odds ratio (95% CrI)**
ALPS-COVID IP	0.91 (0.47 to 1.71)
STUDY 00145514	1.93 (0.76 to 5.94)
COVID ARB	1.95 (0.61 to 9.96)
COVID MED	0.97 (0.17 to 3.69)
	τ = 0.98 (on the log-odds scale; 95% CrI 0.05 – 3.32)
