# Supplementary material for: Losartan in hospitalized patients with COVID-19 in North America: An individual participant data meta-analysis
Source: Medicine (Baltimore). 2023 Jun 9;102(23):e33904. doi: 10.1097/MD.0000000000033904 (PMC10256351; doi:10.1097/MD.0000000000033904)
Supplement: Supplementary file 10 [file medi-102-e33904-s010.pdf]

Figure S1. Estimated Day 13-16 Mortality Rate in Subgroups Under Both Control and ACEi/ARB

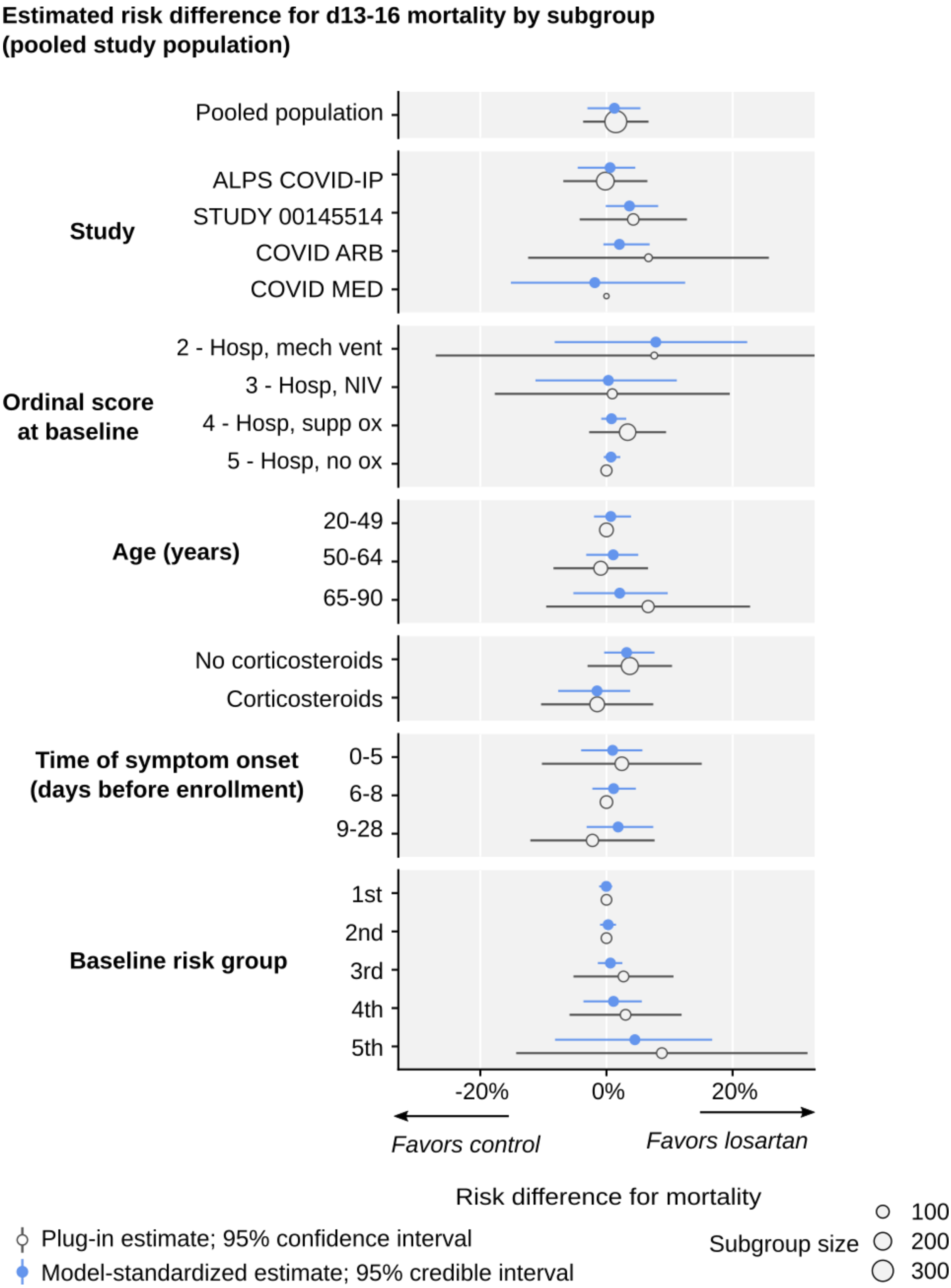

Plug-in estimates are accompanied by 95% confidence intervals; model-standardized estimates are posterior medians with 95% credible intervals. Risk groups are derived from expected outcomes for each patient under control in the model with treatment-covariate interactions.
