# Supplementary material for: Losartan in hospitalized patients with COVID-19 in North America: An individual participant data meta-analysis
Source: Medicine (Baltimore). 2023 Jun 9;102(23):e33904. doi: 10.1097/MD.0000000000033904 (PMC10256351; doi:10.1097/MD.0000000000033904)
Supplement: Supplementary file 11 [file medi-102-e33904-s011.pdf]

Figure S2. Posterior Predictive Check of Day 13-16 Ordinal Score by Site

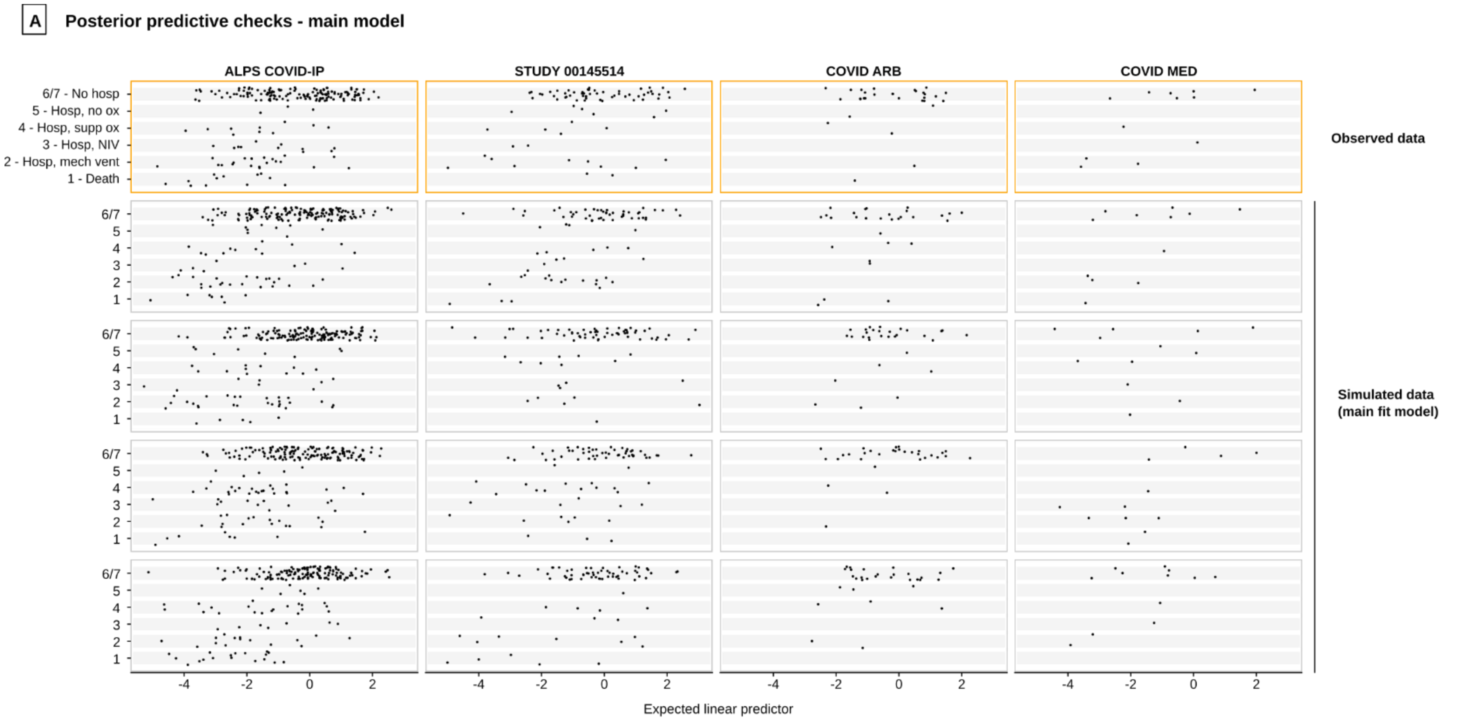

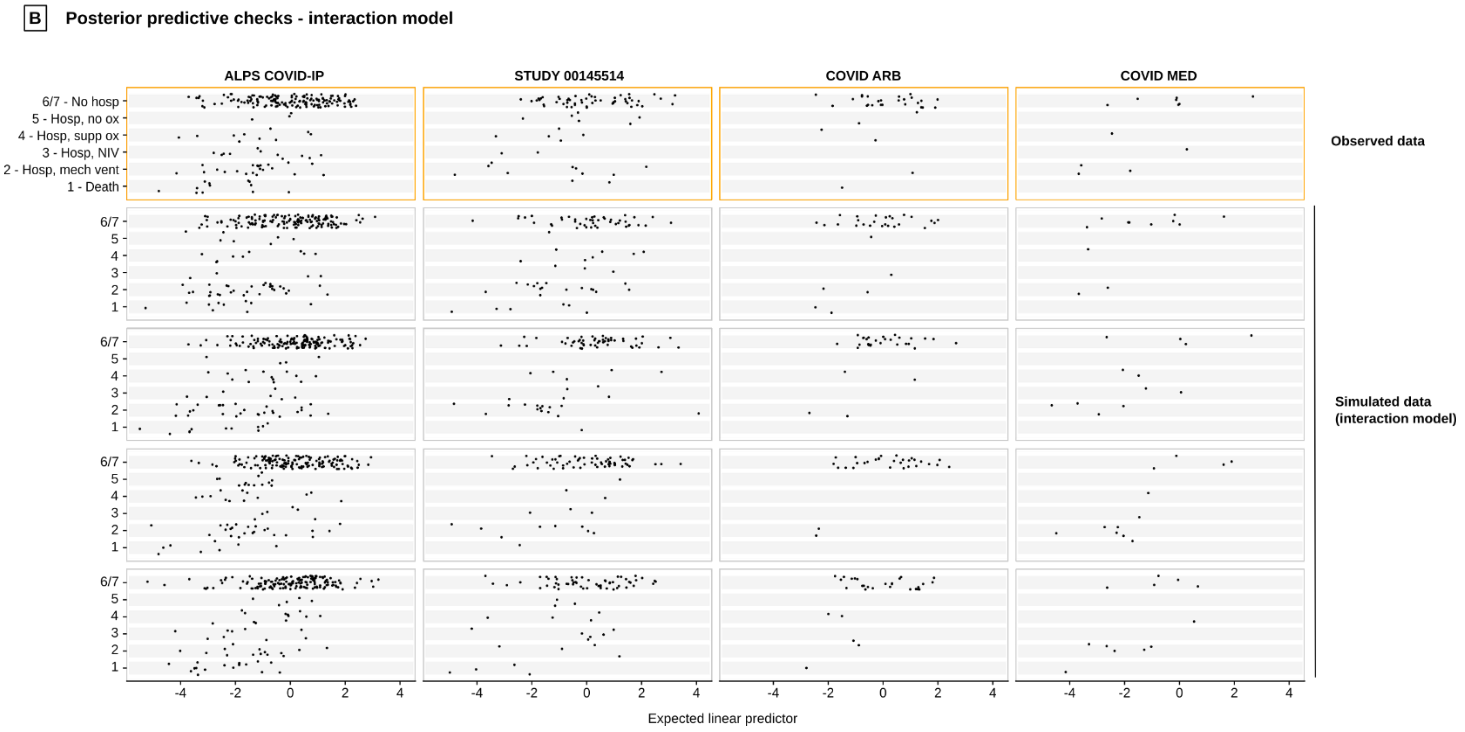

Comparing observed data (orange subplots) with posterior draws (grey subplots) under both the main analysis model with no treatment-covariate interactions (**A**) and the model including treatment-covariate interactions (**B**). Data and posterior draws are stratified by study.
