# Supplementary material for: Losartan in hospitalized patients with COVID-19 in North America: An individual participant data meta-analysis
Source: Medicine (Baltimore). 2023 Jun 9;102(23):e33904. doi: 10.1097/MD.0000000000033904 (PMC10256351; doi:10.1097/MD.0000000000033904)
Supplement: Supplementary file 12 [file medi-102-e33904-s012.pdf]

Table S8. Estimated Predictive Performance of the Models

	Model	Estimated leave-one-out log predictive density (standard error)	Estimated difference in leave-one-out log predictive densities (standard error)
Day 7	main effect model	-408.0 (17.6)	-3.5 (1.9)
	interaction model	-411.4 (17.9)	
Days 13-16	main effect model	-306.6 (20.9)	-2.3 (2.4)
	interaction model	-308.9 (21.2)	
Days 28-30	main effect model	-185.2 (20.2)	-4.0 (2.0)
	interaction model	-189.2 (20.8)	

Estimates of predictive performance were generated using the “loo” and “loo_compare” functions from the R package “loo”, version 2.4.1. These estimates use only one imputation of missing covariate data, and do not take into account correlations among the responses of individuals from the same study.
