# Supplementary material for: Losartan in hospitalized patients with COVID-19 in North America: An individual participant data meta-analysis
Source: Medicine (Baltimore). 2023 Jun 9;102(23):e33904. doi: 10.1097/MD.0000000000033904 (PMC10256351; doi:10.1097/MD.0000000000033904)
Supplement: Supplementary file 13 [file medi-102-e33904-s013.pdf]

Supplemental Appendix. Collaborators from the Studies Analyzed

**STUDY 00145514 (University of Kansas Medical Center)**	
Robert N Montgomery, PhD	Department of Biostatistics and Data Science, University of Kansas Medical Center, Kansas City, KS
Usman Nazir, MD	Department of Internal Medicine, University of Kansas Medical Center, Kansas City, KS
Lewis Satterwhite, MD	Department of Internal Medicine, University of Kansas Medical Center, Kansas City, KS
Michael D Kim, PhD	Department of Internal Medicine, University of Kansas Medical Center, Kansas City, KS
Nathan C Bahr, MD	Department of Internal Medicine, University of Kansas Medical Center, Kansas City, KS
Mario Castro, MD, MPH	Department of Internal Medicine, University of Kansas Medical Center, Kansas City, KS
Nathalie Baumlin, MBA	Department of Internal Medicine, University of Kansas Medical Center, Kansas City, KS
**COVID ARB (Sharp)**	
George Sakoulas, MD	Department of Pediatrics, University of California San Diego School of Medicine, La Jolla, CA
**ALPS-COVID IP (University of Minnesota)**	

Sarah M Lindberg, MPH	Division of Biostatistics, University of Minnesota, Minneapolis, Minnesota
Helen T Voelker, BA	Division of Biostatistics, University of Minnesota, Minneapolis, Minnesota
**COVID MED (Bassett)**	
Paul Jenkins, PhD	Bassett Research Institute, Cooperstown, NY
James Wheeler, MD	Goshen Health, Goshen, IN
G. Matthew Vail, MD	Reid Health, Richmond, IN
Erik Riesenfeld, MD	Bassett Medical Center, Cooperstown, NY
Umesha Boregowda, MD	Bassett Medical Center, Cooperstown, NY
Farah Deshmukh, MD	Bassett Medical Center, Cooperstown, NY
Yuri Choi, MD	Bassett Medical Center, Cooperstown, NY
Azkia Khan, MD	Bassett Medical Center, Cooperstown, NY
